# Transcriptional regulatory network of *WOX11* is involved in the control of crown root development, cytokinin signals, and redox in rice

**DOI:** 10.1093/jxb/erx153

**Published:** 2017-07-11

**Authors:** Wei Jiang, Shaoli Zhou, Qian Zhang, Huazhi Song, Dao-Xiu Zhou, Yu Zhao

**Affiliations:** 1 National Key Laboratory of Crop Genetic Improvement, Huazhong Agricultural University, Wuhan, China; 2 Institute of Plant Sciences Paris-Saclay (IPS2), Université Paris-Saclay, Université Paris-Sud, Orsay, France

**Keywords:** Cytokinin, redox, rice, root development, transcriptional regulation, *WOX11*

## Abstract

The rice root system is mainly composed of post-embryonic shoot-borne roots called crown roots. *WOX11*, encoding a WUSCHEL-related homeobox domain transcription factor, is a key regulator of crown root growth and development in rice (*Oryza sativa*. L). In addition to specifically activating crown root development, *WOX11* is also involved in lateral root initiation, root hair formation, and abiotic stresses. However, the gene regulatory network downstream of *WOX11* remains largely unknown. Here, we studied the transcriptome of *wox11* root tips by RNA-Seq and determined direct WOX11-binding targets by bioinformatic and biochemical analysis. The transcriptomic analysis revealed 664 differentially expressed genes, which covered a wide range of functions related to root development, cytokinin homeostasis/signaling, stress response, and redox metabolic processes. Bioinformatic analysis also revealed that the WOX11-binding motif was distributed over 41% (273/664) of the differentially expressed genes, and was mostly enriched in the promoter and intron regions. We used qRT-PCR and/or *in situ* hybridization to confirm co-expression of some of the *WOX11*-regulated genes in crown root development. We also used electrophoretic mobility shift assay and chromatin immunoprecipitation with anti-WOX11 antibody to validate direct regulation of these genes by WOX11. The analysis identified several genes that acted downstream of WOX11 in controlling crown root formation, cytokinin signaling, stress response, and redox metabolism. This work built a hierarchical regulatory model of *WOX11* in rice crown root development.

## Teaser

Rice *WOX11* plays a regulatory role high in the hierarchy of control of multiple processes such as hormone signaling, stress response, redox, and metabolism during crown root development.

## Introduction

During the past few years, crop improvement has mainly focused on increasing shoot biomass and seed yield (Gonzalez *et al.*, 2009; Xing and Zhang, 2010). The relevance of the root system for food production has often been overlooked. The root system is critical for uptake of nutrients and water from the soil, anchorage, and interaction with symbiotic organisms. Therefore, understanding the mechanism that controls root system patterning and identifying genes responsible for postembryonic root initiation and development could help breeders to increase yield and to improve crop tolerance to abiotic stress (Coudert *et al.*, 2011).

Rice (*Oryza sativa* L.) is the most widely cultivated cereal crop in the world. It has a complex root system structure with several root types, which is different from the dicot model plant Arabidopsis. Crown roots, which initiate from stem nodes or coleoptile sections, constitute the major part of the rice root system and play important roles during growth and environmental adaptation (Xu and Hong, 2013). The biogenesis and development of crown roots are very complex processes. In addition to genetic control, environmental cues and plant hormones affect root development. It is well known that cytokinin signaling and perception are necessary for root development (Mähönen *et al.*, 2006*b*). Cytokinin, perceived by the AHK3/ARR1 and AHK3/ARR12 two-component signaling pathway, acts to control cell differentiation rate in root meristem, and therefore controls root meristem size, root growth, and lateral root formation (Beemster and Baskin, 2000; Dello Ioio *et al.*, 2007; Laplaze *et al.*, 2007). Alteration of expression of cytokinin response regulator genes *RR3*, *RR5*, and *RR6* affects rice root development (Hirose *et al.*, 2007; Cheng *et al.*, 2010). Mutation of *WOODEN LEG* (*WOL*) (a putative two-component histidine kinase) and loss of *ARABIDOPSIS HISTIDINE PHOSPHOTRANSFER PROTEIN6* (*AHP6*), an inhibitor of cytokinin signaling, cause the loss of phloem and vascular cells in the roots (Mähönen *et al.*, 2000, 2006*a*). *CYTOKININ OXIDASE*/*DEHYDROGENASE4* (*OsCKX4*) plays a positive role in crown root formation (Gao *et al.*, 2014). In addition, *crown rootless5* (*CRL5*) promotes crown root initiation through repression of cytokinin signaling (Kitomi *et al.*, 2011). Ubiquitin ligase EL5 maintains the viability of root meristem by influencing cytokinin-mediated nitrogen effects in rice (Mochizuki *et al.*, 2014). The *WUSCHEL*-related h*o*meobo*x* (*WOX*) family gene *WOX11* has been reported to control crown root emergence by directly activating cytokinin signaling (Zhao *et al.*, 2009). Recently, ERF3 was identified as a WOX11-interacting partner, and was shown to enhance WOX11-mediated repression of *RR2* (Zhao *et al.*, 2015). However, it remains unclear how *WOX11* integrates cytokinin signaling to control crown roots formation.

Except for the roles in root development, the function of WOX11 in response to abiotic stress has not been explored until recently. Chen *et al.* (2013) have reported that microcystin-LR (MC-LR) treatment significantly inhibited rice root growth and resulted in a decrease of *WOX11* expression. It has also been shown that *WOX11* responds to many abiotic stresses, such as drought, cold, and high salt (Cheng *et al.*, 2014). In addition, ectopic expression of *WOX11* gene driven by the promoter of *OsHAK16P* encoding a low-K-enhanced K transporter not only led to an extensive crown root system, but also increased total K uptake and grain yield by 24–32% (Chen *et al.*, 2015). Recent results further suggested that *WOX11* contributed to drought resistance by promoting growth of roots and development of root hairs (Cheng *et al.*, 2016). How *WOX11* balances the trade-offs between the need for root growth and resistance to abiotic stress, as well as metabolism, needs to be further understood.

To study the *WOX11* regulatory network, we identified genes that are differentially expressed in *wox11* root *versus* wild-type by RNA-Seq. We found that *WOX11* regulates the expression of about 700 genes in roots, many of which are likely to be the direct targets of WOX11, as demonstrated by analysis with electrophoretic mobility shift assay (EMSA) and chromatin immunoprecipitation (ChIP) assay. *WOX11*-regulated genes are involved in root development, stress response, hormone signaling, and redox metabolism. These results suggest that *WOX11* controls a large regulatory network of genes that may be involved in the formation of crown roots in rice.

## Material and methods

### Plant materials and growth conditions

The rice variety (*Oryza sativa* ssp. *japonica*) used was from the ‘Hwayuong’ (HY) and Zhonghua 11 (ZH11) background. The *wox11* mutant (2A00597 from the mutant library of Pohang University of Science and Technology, South Korea) and overexpressing *WOX11* (*OxWOX11*) transgenic plants were reported by Zhao *et al* (2009).

For seedling growth, seeds of *wox11*, *OsWOX11* (*OW1*, *OW2*) and their corresponding wild type (HY for the *wox11* mutant, ZH11 for *OsWOX11*) were surface-sterilized by 0.15% HgCl_2_, washed with sterilized ddH_2_O eight times, and then germinated in 1/2 MS medium containing 0.8% agar and 3% sucrose at 28 °C (in light) and 24 °C (in dark) with a 14 h light–10 h dark cycle. Root tips of 3–5 mm of 7-day-old seedlings were harvested for RNA extraction.

### RNA-Seq analysis

Total RNA from rice root tips (3–5 mm) of 7-day-old *wox11* mutant and wild type (HY) seedlings were isolated using TRIzol reagent (Invitrogen). The collected root tip region corresponds to the expression domain of *WOX11* (Zhao *et al.*, 2009). RNA libraries were prepared according to the protocol provided by Illumina. Briefly, 4 mg of total RNA was used for mRNA purification and cDNA synthesis. After first and second strand cDNA was synthesized, single ‘A’ nucleotide was added to the blunt-ended cDNA, and indexing adapters (from TruSeq ChIP Sample Preparation kit, Illumina) were ligated subsequently. DNA fragments were enriched by PCR with ten cycles. The amplified DNA fragments were purified and sequenced with the Illumina HiSeq-3000 system. High throughput sequencing resulted in the generation of ~50 million raw reads for each sample. Two independent biological repeats were analysed.

For data analysis, after removing low quality tags with Trimmomatic (version 0.32) (Bolger *et al.*, 2014), clean tags were aligned to the rice genome (RGAP, version 7.0) by TopHat (version 2.0.13). Suites of Cufflinks software were used to assemble transcripts and find differentially expressed genes (fold change>2, *P*<0.05) (Trapnell *et al.*, 2012).

For searching for cytokinin-inducible genes, published data (GSE39429) were used (http://ricexpro.dna.affrc.go.jp/) (Sato *et al.*, 2013). The cytokinin induction ability of all genomic genes was calculated. Fold change was defined as treatment value (experimental group *versus* mock group) divided by pre-treatment value. Genes with fold changes greater than or equal to 2 times were defined as cytokinin-inducible genes. The website http://ricearray.org/ was used for Gene Ontology (GO) enrichment analysis.

Sequences of 1 kb promoter and gene body were downloaded from a website (RGAP, version 7.0), and genes with TTAATGG/C (or reverse complemented) sequence in the promoter or gene body were defined as genes with a WOX11-binding motif.

### RNA extraction and qRT-PCR analysis

Total RNA was extracted from wild type (HY and ZH11), *wox11* mutant, and *OxWOX11* roots using TRIzol reagent (Invitrogen) according to the manufacturer’s instructions. For reverse transcription, 4 μg of total RNA was digested by 1 μl DNase I in a total volume of 10 μl. Oligo dT was conjugated with poly A tail by treatment at 65 °C for 10 min and on ice for 2 min. MLV (reverse transcriptase, Invitrogen) was used for reverse transcription at 37 °C for 1.5 h with RNase inhibitor (Invitrogen) and dithiothreitol. Products were diluted by adding 140 μl ddH_2_O. For real-time PCR analysis, 0.75 μl of RT reactions and 0.25 μM gene-specific primers were mixed with 6.25 μl SYBR Green Master mix in a total volume of 12.5 μl on a 7500 real-time PCR machine (Applied Biosystems) according to the manufacturer’s instructions. The reactions were performed at 95 °C for 10 s, 45 cycles of 95 °C for 5 s, and 60 °C for 40 s. The rice *ACTIN1* gene was used as the internal control. Values represent the means obtained from three independent replicates. Bars represent the standard deviation (SD). The primers are listed in Supplementary Table S1 at *JXB* online.

### RNA *in situ* hybridization

RNA *in situ* hybridization was performed as described previously (Zhao *et al.*, 2009). Briefly, root tips and coleoptile nodes of 4-day-old seedlings were fixed in FAA (50% ethanol, 5% acetic acid and 3.7% formaldehyde) at 4 °C for 24 h, dehydrated in an ethanol series, cleared through a chloroform series, and then embedded in paraffin. Sections of 8–12 μm were mounted on RNase-free glass slides and *in situ* hybridization was then performed using digoxigenin-labeled RNA probes transcribed with either T7 or SP6 transcriptase from pGEM-T plasmids containing part of these genes sequence, which were amplified with gene-specific primers (Supplementary Table S1).

### Chromatin co-immunoprecipitation assay

About 1 g root tips of seedlings 7 days after germination were harvested and cross-linked with 1% formaldehyde for 10 min under vacuum. Chromatin was fragmented by sonication and incubated with the following antibodies: 20 μl WOX11 antibody (reported in Zhao *et al.*, 2015) coupled to protein A beads. Immunoprecipitated chromatin was analysed by qRT-PCR. Enrichment was calculated as a ratio of bound sequence over input. Data are presented as fold change relative to the control. Specific primers were used for qRT-PCR (Supplementary Table S1).

### Electrophoretic mobility shift assay

GST-WOX11 protein was expressed in *E. coli BL21* (DE3) and purified with Glutathione Sepharose 4B (Glutathione Sepharose 4 Fast Flow, GE Healthcare, 17-513-01) according to the manufacturer’s instructions. EMSA was performed using the Light Shift Chemiluminescent EMSA kit (Thermo Scientific, 20148) according to the manufacturer’s instructions.

### Cytokinin and PEG treatment

Seeds were sown and germinated on agar medium. After 10 days, the seedlings were transferred to ultrapure water with or without 10^–5^ M 6-benzylaminopurine (6-BA), or 20% (w/v) PEG6000 (mimicking drought stress) at 28 °C (in light). Total RNA of root tip was extracted after different time courses of treatment and analysed by qRT-PCR with specific primers (Supplementary Table S1).

## Results

### Genome-wide identification of *WOX11* targets in rice crown roots

To study the gene regulatory function of *WOX11*, RNA-Seq analysis was performed from wild type (HY) and *wox11* root tips (Supplementary Fig. S1A). The sequence data displayed a high reproducibility (*R*^2^>0.9759, Supplementary Fig. S1B). Above 95% of the reads were of high quality, of which about 90% aligned well to the rice genome sequence. Above 55% of the aligned reads were unique (Supplementary Table S2). The unique aligned reads were used to calculate the relative abundance of transcripts.

**Fig. 1.**
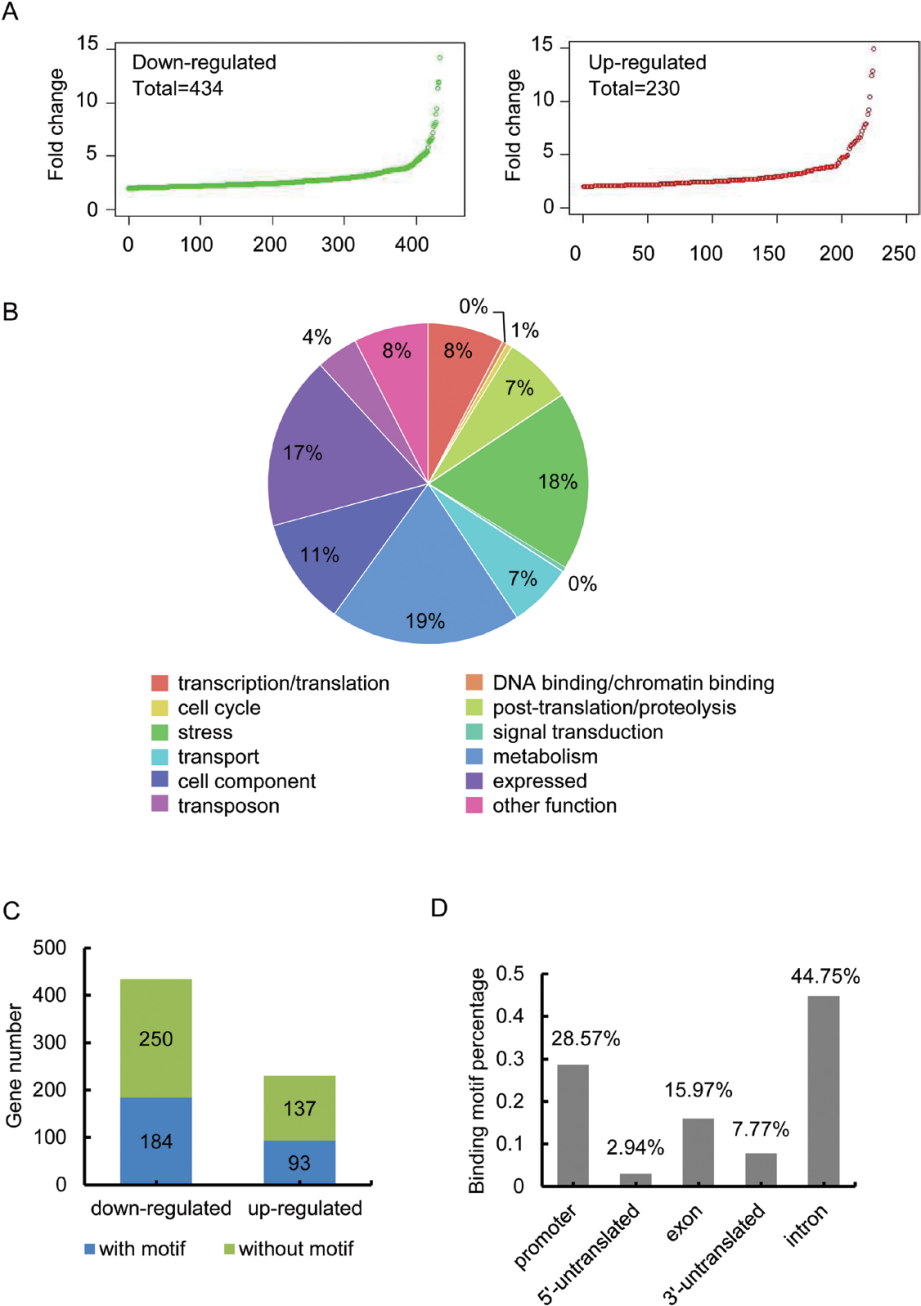
Analysis of genome-wide transcript profile and WOX11 binding motif in the differentially expressed genes. (A) Fold change of 434 down- and 230 up-regulated genes in 7-day-old crown root tips (3–5 mm) of *wox11* mutant relative to wild type (HY). Each abscissa unit corresponds to a unique gene. Green and red circles represent down- and up-regulated genes, respectively. (B) Functional categorization of 667 genes with altered expression in *wox11* crown root by Gene Ontology analysis. Expressed: genes without functional annotation; transport: genes playing roles in intercellular transport. (C) Numbers of genes with WOX11-binding site (TTAATGG/C) in differentially expressed genes. (D) Distribution of WOX11-binding sites in the differentially expressed genes.

The expression levels of 664 genes (434 down-regulated and 230 and up-regulated) changed at least 2-fold in *wox11 versus* wild type (*P*<0.05). About 10% of the genes were differentially expressed more than 5-fold (Fig. 1A). Most of the differentially expressed genes fell into the metabolism, stress, cell components, and signal transduction categories (Fig. 1B), while about 8% of differentially expressed genes had no annotated function. The function of 77 out of 664 (11%) differentially expressed genes has been previously studied. Interestingly, most of these studied genes have an assigned function related either to hormone signaling, in particular cytokinin and auxin, or to gene function associated with root development or stress/metabolism. Next, we asked whether the differentially expressed genes between the wild type and *wox11* roots were directly regulated by *WOX11*. The DNA-binding motif of WOX11 (TTAATGG/C), which has been reported previously, was investigated in these genes (Lohmann *et al.*, 2001; Kamiya *et al.*, 2003; Dai *et al.*, 2007; Zhao *et al.*, 2009, 2015). Sequences of 1 kb upstream from the transcription start site (TSS) to transcription termination sites (TTS) were downloaded from the RGAP website (version 7.0). Genes with the presence of TTAATGG/C sequence in the promoter or genic region were identified as WOX11-binding targets. As shown in Fig. 1C and Supplementary Table S3, 277 (184 down-regulated, and 93 up-regulated) of 667 differentially expressed genes were found to contain the WOX11-binding motif. Of these, 45% of the binding sites are located in introns, 28.6% in the promoter regions (−1 kb to the TSS), 16.0% in exons, and 7.8% in the 3′-untranslated regions of these genes. Only about 3% are located in the 5′-untranslated regions (Fig. 1D). Together, these data indicate that WOX11 preferentially binds to the promoter and/or intron, and regulates genes involved in specific tissues and/or at development stages.

### WOX11 directly controls root development-related genes by binding to their regulatory elements

To assess which of these differentially expressed genes were expressed specifically in root, we analysed their expression profiles in different tissues/organs according to rice microarray data (http://ricexpro.dna.affrc.go.jp/) (Sato *et al.*, 2011). About 52% (199 out of 385) down- and 39% (69 out of 179) up-regulated genes were expressed specifically in roots (Supplementary Fig. S2A). Surprisingly, 81 of those that expressed specifically in root contained WOX11-binding motif and were down-regulated in *wox11* (Supplementary Table S4). Further survey of these genes expression patterns in different regions of root tip revealed that the majority of the putative WOX11 targets were preferentially expressed in the elongation region (EZ) and maturation region (M1) (Supplementary Fig. S2B), where lateral roots and root hairs initiate. The result corroborated the WOX11 function in lateral initiation and root hair development (Zhao *et al.*, 2009; Cheng *et al.*, 2016).

**Fig. 2.**
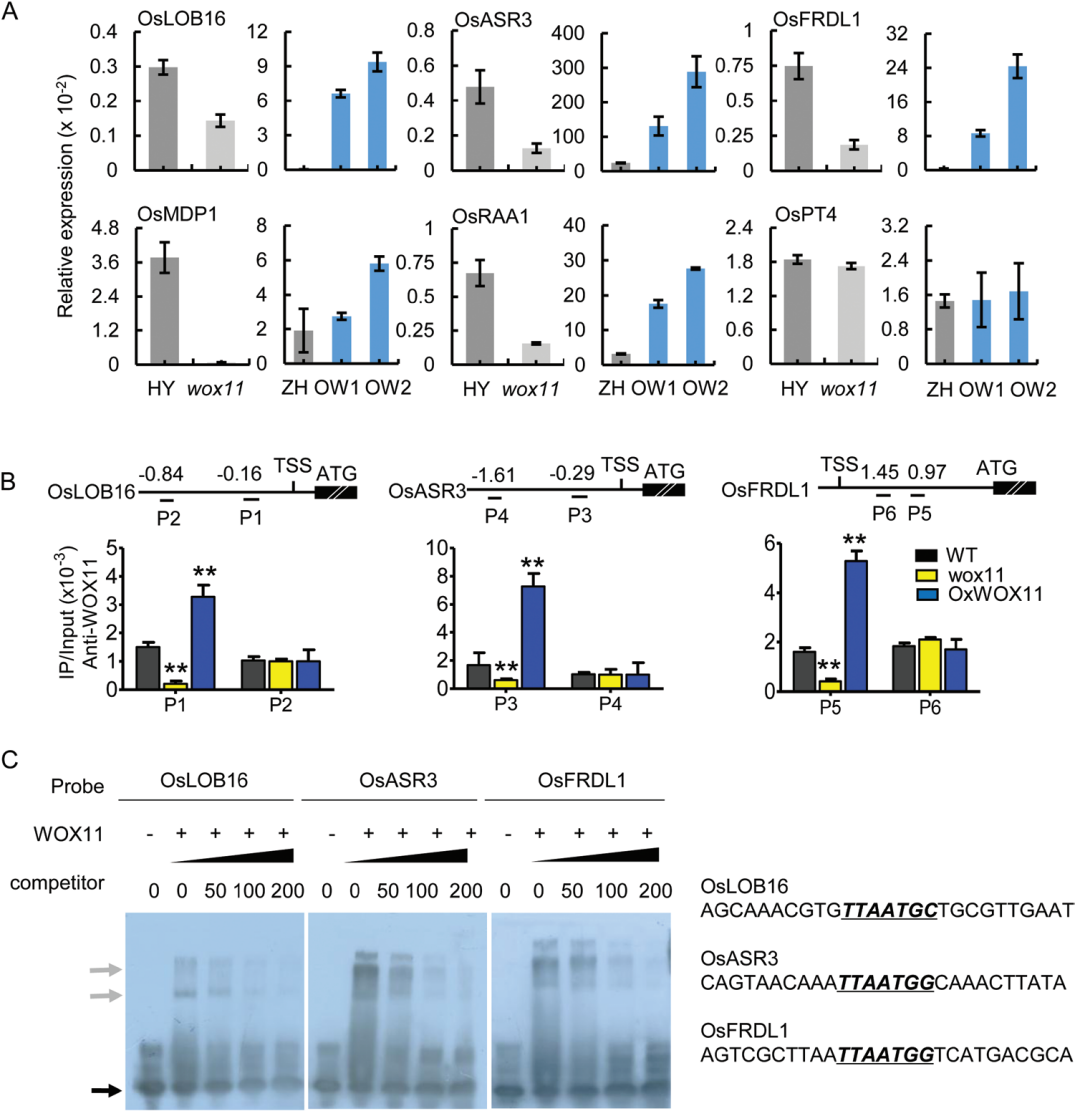
*WOX11* regulates expression of root development-related genes. (A) Validation of transcript levels of root development-related genes in 7-day-old crown root tips (3–5 mm) of *wox11*, *OxWOX11* (*OW1*, *OW2*), and their corresponding wild type plants. Relative transcript levels to *ACTIN1* are represented as the *y*-axis values. Data represent the means of three biological replicates. Values are means±standard deviation (SD) (*n*≥6). (B) ChIP-qPCR assay confirmation of *in vivo* binding of WOX11 to root development-related genes in 7-day-old crown root tips (3–5 mm). Upper panel, diagrams of the indicated binding loci. The transcribed regions (thick lines), transcription start sites (TSS), translation start sites (ATG), and primer sets (P1 to P6) used in ChIP experiments are indicated. Numbers indicate distance (kb) of primers from TSS. Lower panel, ChIP assays with anti-WOX11 in wild type (HY), *wox11* and *OxWOX11* crown roots. Data represent the means of three biological replicates. Values are means±standard deviation (SD) (*n*≥6), and asterisks represent statistically significant difference (*t*-test, *P<0.05, **P<0.01). (C) Recombinant WOX11 protein binds to biotin-labeled oligonucleotides, which contain the WOX11-binding site. The upper arrow indicates the shifted band and the lower arrow indicates free probes.

To test whether the differentially expressed genes identified in our transcriptome analysis were directly regulated by WOX11 during crown root development, we selected a subset of six genes (*OsLOB16*, *OsASR3*, *OsFRDL1*, *OsMDP1*, *OsRAA1*, and *OsPT4*) for which functions have been reported. They all contained the WOX11-binding motif and were down-regulated in *wox11*. Firstly, we analysed their expression patterns in root tip by qRT-PCR in *WOX11* transgenic lines and wild type plants. The results showed their significant down-regulation in *wox11* roots and great up-regulation in *OxWOX11* (*WOX11* overexpressing plants) roots (Fig. 2A), suggesting that WOX11 might act as a direct activator of these genes in roots. Two of the genes, *OsASR3* (*Os02g33820*) and *OsFRDL1* (*Os03g11734*), were reported to be most abundant in rice roots (Yokosho *et al.*, 2009; Joo *et al.*, 2013). *OsLOB16* (*Os02g57490*), which is homologous to *OsCRL1* (*Os03g05510*), but more closely related to Arabidopsis *LBD16* (a downstream gene of Arabidopsis *WOX11*/*12* in root cell fate control) (Liu *et al.*, 2014), was expressed in root epidermis (Supplementary Fig. S3). These results further suggested that these genes were potential direct targets of WOX11. Next, we tested whether WOX11 directly bound to the *cis*-elements (TTAATGG/C) of these putative targets *in vivo*. We performed chromatin immunoprecipitation (ChIP) coupled to qPCR by using anti-WOX11 antibody of chromatin extracted from root tips of wild type, *wox11*, and *OxWOX11*. ChIP-PCR analysis revealed that in wild type roots, the WOX11 binding was enriched in the P1 region (from −0.16 kb to TSS) of *OsLOB16*, the P3 region (0.29 kb to TSS) of *OsASR3*, and the P5 region (1.45 kb to TSS) of *OsFRDL*. The binding was much reduced in the *wox11* mutant (Fig. 2B). We further found that oligonucleotides containing the ‘TTAATGG/C’ sequence motif in the promoter of these three genes bound to WOX11 in EMSAs, and increasing molar excesses of unlabeled fragment (competitor) inhibited the binding (Fig. 2C).

Taken together, these results strongly demonstrate that WOX11 directly activates transcription of these three genes via binding to the DNA motif in rice crown roots.

### Cytokinin homeostasis/signaling pathway may be altered in *WOX11* transgenic roots

**Fig. 3.**
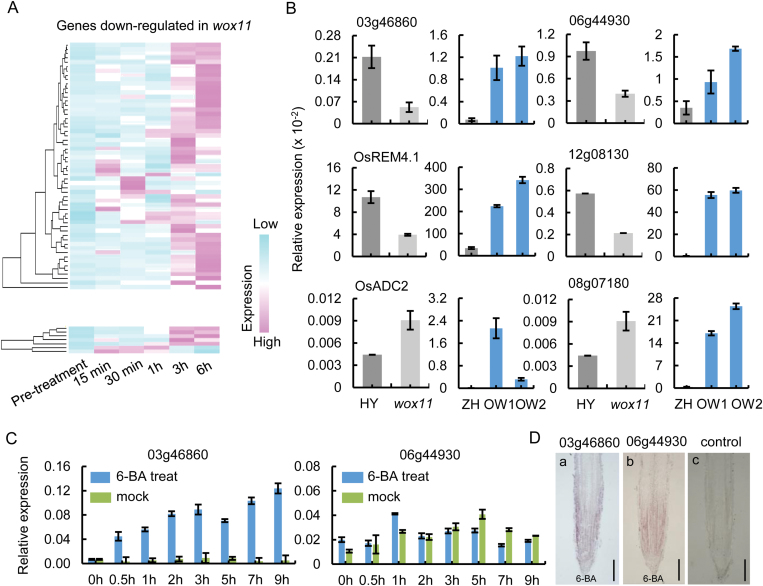
*WOX11* modulates expression of cytokinin-induced genes. (A) Relative transcript levels of 64 identified differentially expressed genes that are cytokinin-inducible. (B) Validation of cytokinin-responsive gene expression in crown root tips (3–5 mm) of 7-day-old wild type (HY and ZH11), *wox11* mutant, and *OxWOX11*(*OW1*, *OW2*). (C) Induction kinetics of two responsive genes to exogenous cytokinin (6-BA). The transcript levels in crown root tips (3–5 mm) of 10-day-old light-grown wild type (ZH11) seedlings treated with 6-BA for the indicated times were plotted as the relative expression (fold) of water-treated seedlings during the same durations. The PCR signals in (B) and (C) were normalized with those of the *ACTIN1* transcripts. Data represent the means of three biological replicates. Values are the means±standard deviation (SD). (D) *In situ* hybridization detection of two genes from (C) with anti-sense probes showing transcription induced by cytokinin in 10-day-old crown root. Scale bars: 200 μm.

Previous studies demonstrated that *WOX11* was induced by exogenous cytokinin and that WOX11 directly suppressed the expression of *RR2*, a type-A cytokinin response factor (Zhao *et al.*, 2009). The Gene Ontology (GO) term-assessed cytokinin pathway was enriched in the WOX11-regulated transcriptome (Supplementary Table S5). We found that 10% (64/664) of differentially expressed genes were responsive to exogenous cytokinin treatment (http://ricexpro.dna.affrc.go.jp/;Sato *et al.*, 2013; Fig. 3A), and notably, 27 of the 64 cytokinin-responsive genes contained the WOX11-binding motif (Supplementary Table S5). This included *Os08g07180* (*O*-glucosyltransferase 2), whose orthologous gene in maize metabolizes active cytokinin (Martin *et al.*, 2001). Analysis of six genes by qRT-PCR confirmed that five showed significant down-regulation in *wox11* and up-regulation in *OxWOX11* roots, while the transcript level of *OsADC2* (*Os04g01690*) increased in both *wox11* and *OxWOX11* roots (Fig. 3B). In order to determine whether these genes were responsive to exogenous cytokinin, wild type (ZH11) 7-day-old seedlings were transferred to media containing 10^–5^ M 6-benzylaminopurine (6-BA). The roots were harvested for RNA extraction at 0, 0.5, 1, 2, 3, 5, 7, and 9 h after treatment. As expected, *Os03g46860* and *Os06g44930* were highly induced by cytokinin after treatment (Fig. 3C). Additionally, *in situ* hybridization also showed that *Os03g46860* and *Os06g44930* were induced by cytokinin in roots (Fig. 3D). Therefore, activation of these genes by *WOX11* may be relevant to establishment or maintenance of cytokinin signaling/homeostasis in rice root development.

### WOX11 regulates expression of genes involved in stress resistance

**Fig. 4.**
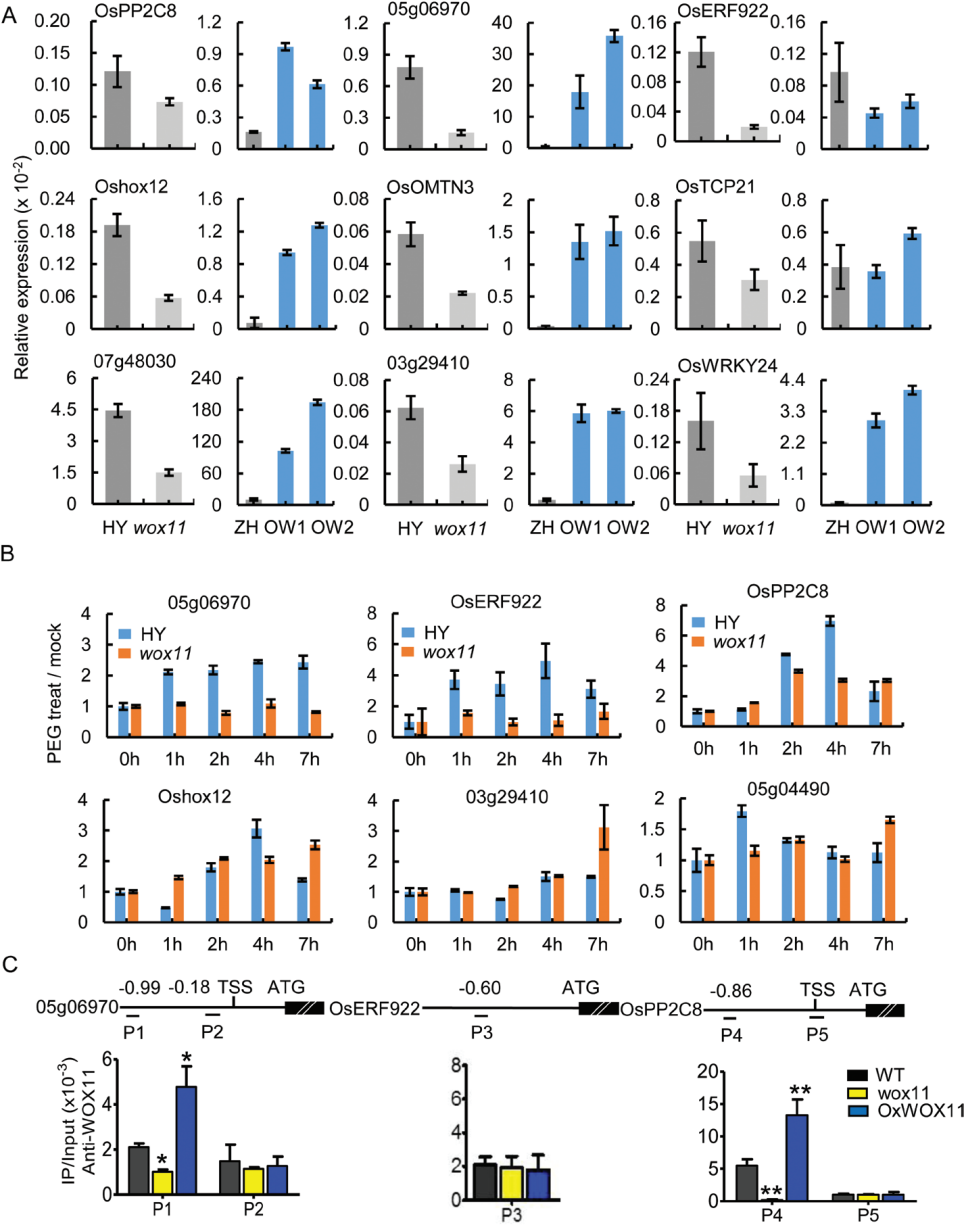
*WOX11* is directly involved in stress-related gene expression. (A) Transcript levels of stress-related genes in crown root tips (3–5 mm) of 7-day-old wild type (HY and ZH11), *wox11*, and *OxWOX11* (*OW1*, *OW2*) plants. (B) Time course of transcript responses to mock and 20% PEG treatment (0, 1, 2, 4, or 7 h) in 10-day-old crown root tips of wild type (HY) and *wox11* assayed by qRT-PCR. The PCR signals in (A) and (B) were normalized with those of the *ACTIN1* transcripts. Values are the means±SD from three biological replicates. (C) ChIP-PCR analysis using anti-WOX11 antibody of crown root tips (3–5 mm) of 7-day-old wild type (HY), *wox11*, and *OxWOX11*. Upper panel, diagrams of the indicated loci. The transcribed regions (thick lines), transcription start site (TSS), translation start site (ATG), and primer sets (P1 to P5) used in the ChIP experiments are indicated. Numbers indicate distance (kb) from TSS. Lower panel, ChIP assays with anti-WOX11 antibody in wild type, *wox11*, and *OxWOX11* root tips. Data represent the means of three biological replicates. Error bars represent standard deviation (SD) and significant difference between different genotypes is indicated by asterisks (*P<0.05, **P<0.01, *t*-test).

RNA-Seq analysis also showed that 18% of differentially expressed genes were involved in response to stress (Fig. 1B). We further analysed the expression of the stress-responsive genes that were down-regulated in *wox11* mutant and contained WOX11-binding motif (Supplementary Table S6). We confirmed that 6 out of the 8 analysed genes were up-regulated by *WOX11* under normal growth conditions (Fig. 4A), which is consistent with the recent results that *WOX11* is involved in abiotic stress (Cheng *et al.*, 2016). To study whether WOX11 is required for stress induction of these genes, wild type and *wox11* plants were treated with 20% polyethylene glycol (PEG) as a water stress, and roots of the treated plants were harvested for transcript analysis by qRT-PCR. The results showed that *Os05g06970* and *OsERF922* (*Os01g54890*) were responsive to PEG only in wild type (Fig. 4B), indicating that the induction of the two genes by PEG stress required *WOX11*. To investigate whether the two genes were directly controlled by WOX11, ChIP-qPCR was performed. Only the *Os05g06970* P1 region (from −0.99 kb to TSS) was found to be associated with WOX11 in the wild type (Fig. 4B). On the other hand, the induction of *OsPP2C8* (*Os01g46760*), *Oshox12* (*Os03g29410*), and *Os05g04490* by PEG was observed in both wild type and *wox11* root tips (Fig. 4B), indicating that the induction of these genes by PEG was not under the control of *WOX11*. It was reported that *OsPP2C8* (*Os01g46760*) encodes a putative protein phosphatase 2C and most of the orthologous genes in Arabidopsis were reported to play important roles in drought stress (Yoshida *et al.*, 2006). ChIP-qPCR assays confirmed that WOX11 bound to this gene (Fig. 4C), suggesting that WOX11 might be involved in the regulation of the genes that were independent of the PEG stress response.

### WOX11 regulates gene transcription related to redox metabolic pathway

**Fig. 5.**
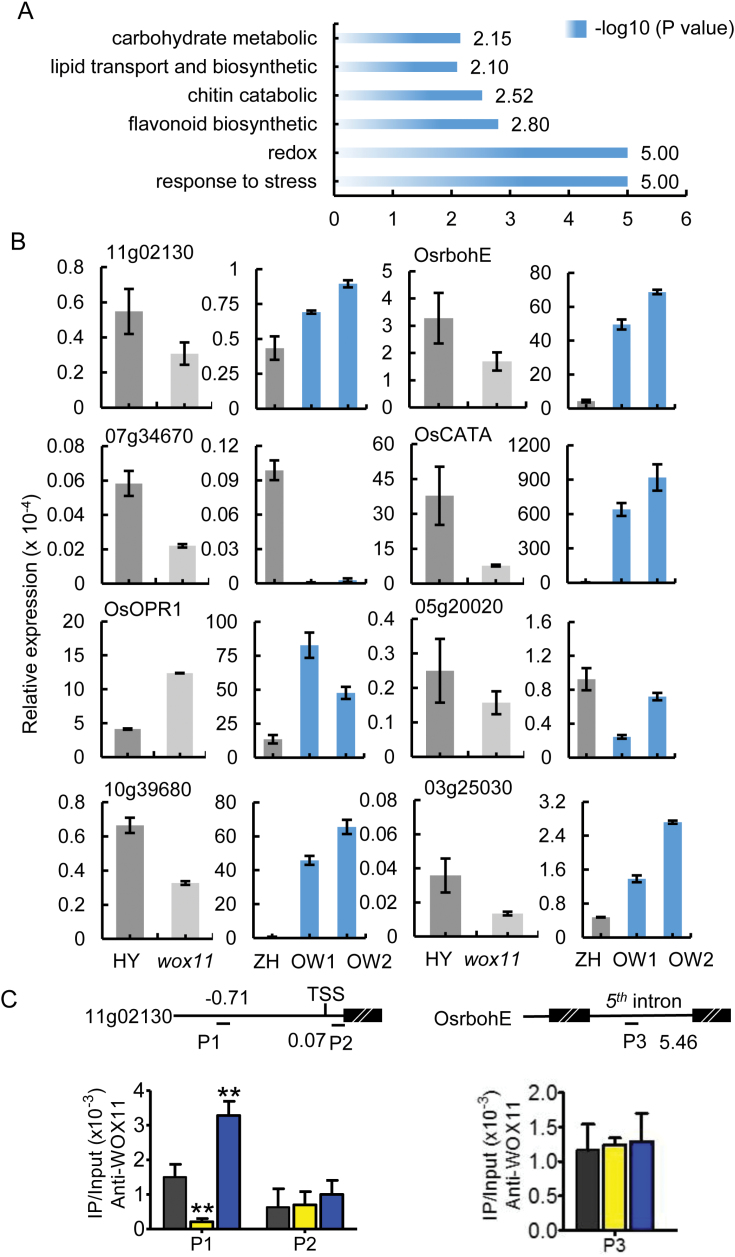
*WOX11* is involved in controlling transcripts of metabolism-related genes. (A) Metabolism GOs as indicated are enriched in the differentially expressed genes. Values of the *x*-axis are −log*P*. (B) qRT-PCR validation of metabolism-related genes regulated by *WOX11* in 7-day-old crown root tips (3–5 mm). The PCR signals were normalized with those of the *ACTIN1* transcripts. Values are means±SD from three biological replicates. (C) ChIP-PCR analysis using anti-WOX11 antibody of 7-day-old wild type (HY), *wox11*, and *OxWOX11* crown root tips (3-5mm). Upper panel, diagrams of the indicated loci. The transcribed regions (thick lines), transcription start site (TSS) and primer sets (P1 to P3) used in the ChIP experiments are indicated. Numbers indicate distance (kb) from TSS. Lower panel, ChIP assays with anti-WOX11 antibody. Data represent the mean of three biological replicates. Values are means±SD. Significant difference is indicated by asterisks (*P<0.05, **P<0.01, *t*-test).

To identify other possible biological processes or pathways that were changed in *wox11* mutant, Gene Ontology (GO) enrichment was performed using a false discovery rate adjusted *P*≤0.05 as the cutoff. Our analysis revealed that 19% of the differentially expressed genes were involved in redox and lipid/carbohydrate metabolic processes (Fig. 5A). qRT-PCR confirmed that the transcript levels of eight selected genes were altered in *wox11* and *OxWOX11* transgenic roots compared with the wild type (Fig. 5B). Of these genes, *OsCATA* (*Os02g02400*) is a well-documented gene that catalyses the decomposition of H_2_O_2_ into oxygen and water (Ye *et al.*, 2011). *OsrbohE* (*Os08g35210*), which encodes an *Oryza sativa* plasma membrane NADPH oxidase, a major enzyme that produces reactive oxygen species (ROS) in plant cells under normal growth and stress conditions (Yoshiaki *et al.*, 2005; Liu *et al.*, 2012*b*; Wang *et al.*, 2016), was also detected among the potential *WOX11* target genes (Supplementary Table S7). *Os11g02130*, which encodes a putative oxidation–reduction enzyme (Supplementary Table S7), was confirmed to be a WOX11 direct target by ChIP-qPCR assay (Fig. 5C). These findings indicated that knock-out of *WOX11* may subsequently activate ROS production in roots. However, effects of redox or lipid/carbohydrate metabolism on crown root formation await further study.

## Discussion

*WOX11* has been shown to regulate rice root development, cytokinin signaling, and resistance to abiotic stress (Zhao *et al.*, 2009, 2015; Cheng *et al.*, 2014, 2016). The present data obtained by using transcript profiling, qRT-PCR, EMSA, and ChIP analysis expanded our understanding of *WOX11* function in gene regulation and its regulatory network controlling crown root development. Our results suggest that WOX11 is required for the expression of genes of several different pathways, including root development (Fig. 2), cytokinin signaling (Fig. 3), stress (Fig. 4), and redox (Fig. 5; Supplementary Table S6). Of these WOX11 target genes, *OMTN3* (*Os12g41680*), a NAC transcription factor gene that was found to be expressed in root meristem and crown root initials in the present study (Supplementary Fig. S3), was previously reported to contribute to cold resistance (Fang *et al.*, 2014). *OsERF922* (*Os01g54890*) and *OsTCP21* (*Os07g48030*) were previously found to negatively modulate plant responses to salinity, pathogen and cold stress, respectively (Liu *et al.*, 2012*a*; Wang *et al.*, 2014). *Os01g61080* (*OsWRKY24*) was previously shown to enhance resistance to salt and drought mediated by the cross-talk of gibberellic acid and abscisic acid signaling pathways under stressful conditions (Zhang *et al.*, 2009). These observations together indicate that WOX11 functions as a higher hierarchical transcriptional regulator of key genes involved in responses to different stresses. In addition, our data revealed that WOX11 is also involved in the regulation of lipid/carbohydrate metabolism genes (*Os10g39680* and *Os03g25030*) (Fig. 5B), suggesting that a wide spectrum of genes are regulated by WOX11, which may be potentially related to root development.

It has been determined that *WUSCHEL*-related homeobox transcription factors recognize the DNA sequence TTAATGG/C *in vitro* (Lohmann *et al.*, 2001; Kamiya *et al.*, 2003; Dai *et al.*, 2007; Zhao *et al.*, 2009; Yadav *et al.*, 2011). Our data indicated that approximately 34% of the differentially expressed genes in *wox11* contained at least one WOX-binding motif in the promoter and intron regions (Supplementary Table S3), which suggests that they could be the direct target genes of WOX11. However, these genes were not particularly enriched in WOX-binding motifs compared with all the rice genes. This might suggest that additional elements present in these genes may be involved in WOX11 targeting. Another possibility may be that other transcription factors binding to these genes may interact and enhance WOX11 targeting to these genes in developing roots, such as the case of the interaction between WOX11 and ERF3 required for the dynamic regulation of *RR2* during crown root initiation and development (Zhao *et al.*, 2015). Alternatively, the chromatin state may also play a role to selectively facilitate the binding of WOX11 to only a subset of genes that contain the WOX-binding sites. Our results also revealed that WOX11 bound to some binding motifs but not all (Fig. 4C), suggesting that sequence context may determine binding specificity. However, a comparison of WOX11-binding sites of several WOX11-regulated genes did not reveal any consensus sequences around the TTAATGG/C core. Shape analysis of the *cis*-element sequence that binds homeodomain transcription factors has revealed that the width of the minor groove determines the binding specificities (Slattery *et al.*, 2011). Future studies of genome-wide WOX11-binding pattern along with sequence and shape of WOX11-binding sites may provide insight into WOX11-binding specificity and transcriptional modulation.

Plants being sessile in nature are often challenged by various abiotic stresses including water supply, salinity, and nutrient availability. Plant roots serve as the major interface between the plant and various biotic and abiotic factors in the soil environment and are more sensitive to abiotic stress. Many reports suggested that the redox process might be important for plant abiotic stress resistance and root development (Chen and Xiong, 2005; Baltruschat *et al.*, 2008). Our previous study also demonstrated that *WOX11* was involved in abiotic stress, especially drought (Cheng *et al.*, 2014, 2016). This study revealed that many differentially expressed genes were related to redox, including six genes encoding peroxidase precursors (*Os05g04490*, *Os05g06970*, *Os07g34670*, *Os07g48030*, *Os07g48060*, *Os11g02130*), two genes encoding catalase (CAT) isozyme (*Os02g02400*, *Os03g03910*), and one encoding NADPH oxidase *OsrbohE* (*Os08g35210*), among which *Os02g02400* and *OsrbohE* (*Os08g35210*) were most significantly changed in *wox11* and *OxWOX11* roots (Fig. 5, Supplementary Table S7). Their repression in the *wox11* mutant may be related to enhancing sensitivity to abiotic stress and higher ROS production. This suggests that redox may be important during crown root development by responding to stress conditions.

Besides, some of the WOX11 targets (e.g. *Os0506970*, *OsERF922*) were rapidly induced by PEG6000, while the induction of others (*Os03g29410*, *OsPP2C8*) was slower. A similar phenomenon was observed in cytokinin treatment experiments. These observations suggest that WOX11, as a highly hierarchical regulator, may take part in both fast and hysteretic responses of gene expression to both cytokinin and PEG6000 treatment.

**Fig. 6.**
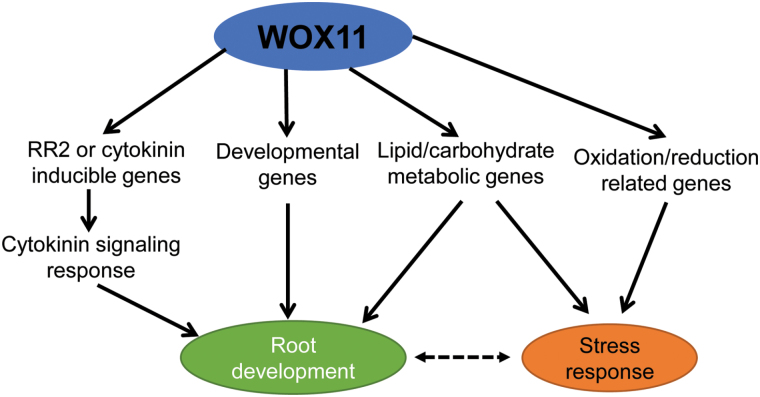
Hypothetical gene regulatory network regulated by *WOX11* in rice root development and stress response. (This figure is available in color at *JXB* online.)

In summary, our results demonstrated that WOX11 was required for the regulation of genes involved in multiple pathways during rice crown root development. Our data suggest a high hierarchical regulatory function of WOX11 in a gene expression network regulating root developmental and stress-responsive processes (Fig. 6). WOX11 not only directly activates genes associated with root development and enzymes involved in redox and carbon metabolism pathways, but also controls the expression of other transcription factors that, in turn, regulate downstream genes of different pathways. Our results identifying WOX11 regulatory pathways will provide insights into the biological function of WOX11 in rice root development and approaches for improving root system development, abiotic resistance, hormone signals, and metabolic processes in the future.

## Supplementary data

Supplementary data are available at *JXB* online.

Fig. S1. Determination of *WOX11* expression level and reproducibility of RNA-seq repeats in *wox11* and wild type (HY) root tips.

Fig. S2. Transcript profiles of the dysregulated genes in the indicated tissue/organ and root tip region in *wox11*.

Fig. S3. Detection of transcripts of two representative differentially expressed genes in root tip and initialing crown roots.

Table S1. Primers used in this study.

Table S2. RNA-Seq reads and analysis data of *wox11* mutant and wild type (HY).

Table S3. WOX11-binding motif numbers in the down- or up-regulated genes.

Table S4. Root-specific differentially expressed genes in *wox11* root tips.

Table S5. Cytokinin-inducible genes regulated by *WOX11.*

Table S6. Stress-related differentially expressed genes

Table S7. Metabolism enrichment GO.

## Accession numbers

Sequence data from this article can be found in the Rice Genome Annotation Project website (http://rice.plantbiology.msu.edu/) under the following accession numbers: *WOX11*, Os07g48560; *OsLOB16*, Os02g57490; *OsASR3*, Os02g33820; *OsFRDL1*, Os03g11734; *OsMDP1*, Os03g08754; *OsRAA1*, Os01g15340; *OsPT4*, Os04g10750; *OsADC2*, Os04g01690; *OsERF22*, Os01g54890; *OsPP2C8*, Os01g46760; *OsOMTN3*, Os12g41680; *OsTCP21*, Os07g05720; *Oshox12*, Os03g10210; *OsrbohE*, Os08g35210; *OsCATA*, Os02g02400; *REM4.1*, Os07g38170; *OsWRKY24*, Os01g61080 and *OsOPR1*, Os06g11290. The RNA-Seq described in this paper have been deposited into the National Center for Biotechnology Information databases (GSE84933).

## Author contributions

WJ, SZ, and YZ designed the experiment. WJ, SZ, QZ, and HS performed the experiments. WJ, SZ, and YZ analysed the data. D-XZ, WJ, and YZ wrote the article.

## Supplementary Material

Supplementary_Figures_S1_S3_Tables_S1_S7
