# Supplementary material for: Transcriptional regulatory network of WOX11 is involved in the control of crown root development, cytokinin signals, and redox in rice
Source: J Exp Bot. 2017 Jul 11;68(11):2787–98. doi: 10.1093/jxb/erx153 (PMC5853245; doi:10.1093/jxb/erx153)
Supplement: Supplementary_Figures_S1_S3_Tables_S1_S7 [file erx153_suppl_supplementary_figures_s1_s3_tables_s1_s7.pdf]

Supplemental Data:

Fig. S1 Determination of *WOX11* expression level and reproducibility of RNA-seq repeats in *wox11* and wild type (HY) root tips.

Fig. S2 Transcript profiles of the dysregulated genes in the indicated tissue/organ and root tip region in *wox11*. LF, leaf; ST, stem; RO, root; AN, anther; PI, pistil; OV, ovary; EN, endosperm; RC, root cap; DZ, division zone; EZ, elongation zone; M1, M2, M3, M4, and M5; different region in maturation zone.

Fig. S3 Detection of transcripts of two representative differentially expressed genes in root tip (**a**, **e**) and initialing crown roots (**c**). Probes used are antisense in **a, c** and **e**, sense in **b, d** and **f**. Bars = 200 μm.

Table S1. Primer used in this study.

Table S2. RNA-Seq reads and analysis data of *wox11* mutant and wild type (HY)

Table S3. WOX11-binding motif numbers in the down- or up-regulated genes.

Table S4. Root-specific differentially expressed genes in *wox11* root tips.

Table S5. Cytokinin-inducible genes regulated by *WOX11*.

Table S6. Stress related differentially expressed genes

Table S7. Metabolism enrichment GO

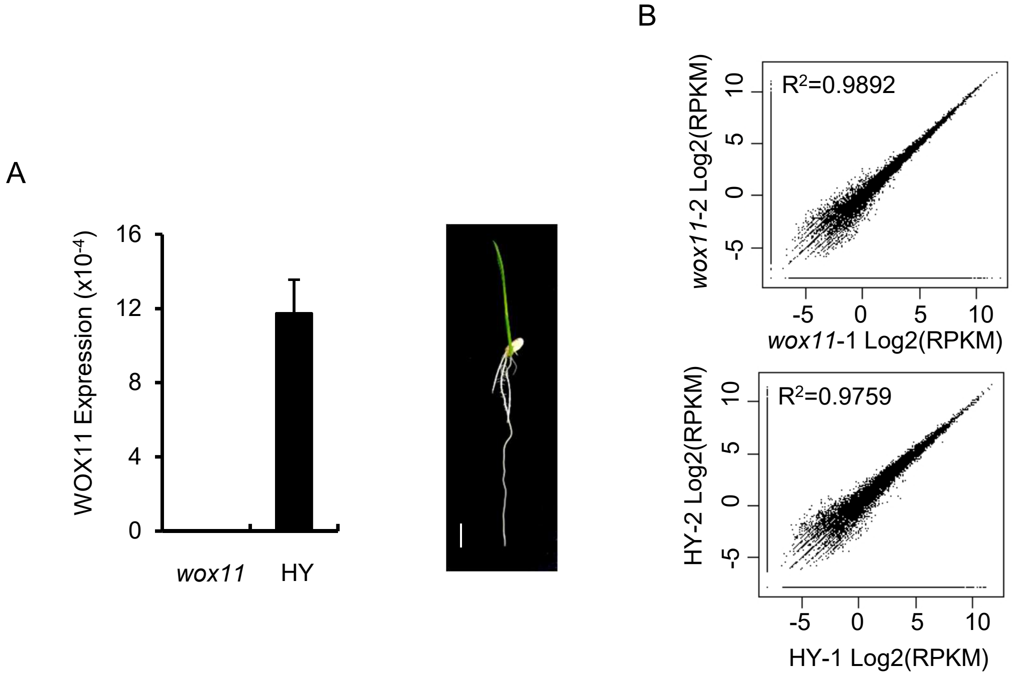

Supplemental Fig.1 Determination of *WOX11* expression level and reproducibility of RNA-seq repeats in *wox11* and wild type root tips. (A) left panel, qRT-PCR examination of *WOX11* expression in *wox11* and wild type (HY). Right panel shows the 7 days seedling of which we harvested the 3-5 mm root tip for RNA-seq. Bar=1cm **(B)** Two-wise scatter plots of RNA-seq reads between two biological repeats. Each point represent one gene locus. RPKM, Reads Per Kilobase per Million reads. Correlation coefficient R^2 indicated.

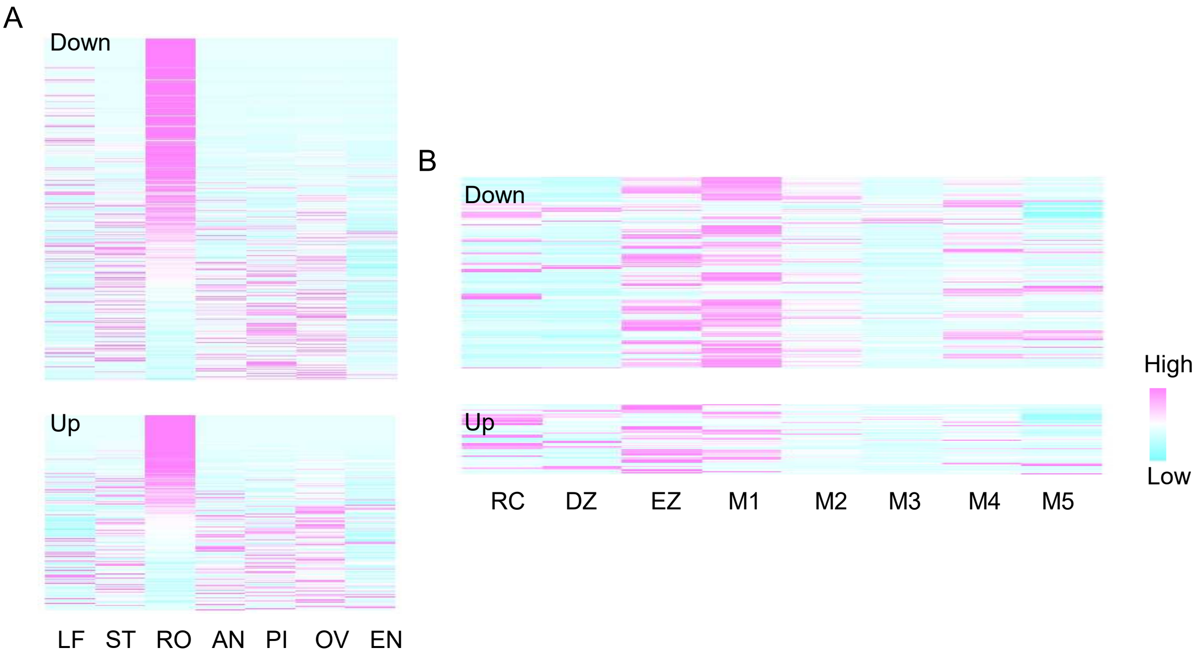

Supplemental Fig.2 Transcription profiles of expression dysregulated genes in the indicated tissue/organ and root tip region in *wox11*. (A) Heat map of transcription profiles in the indicated tissue or organ of expression dysregulated genes in *wox11*. LF, leaf; ST, stem; RO, root; AN, anther; PI, pistil; OV, ovary; EN, endosperm. **(B)** Heat map profile of transcripts of root specific expressed genes regulated by *WOX11* in different regions of root tip, RC, root cap; DZ, division zone; EZ, elongation zone; M1, M2, M3, M4, and M5; different maturation zone.

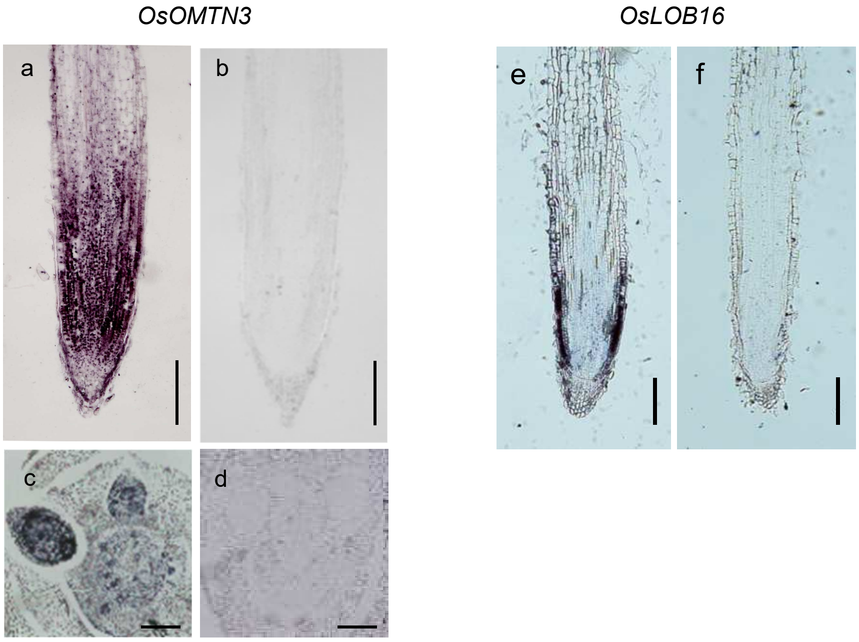

Supplemental Fig. S3 Detection of transcripts of two representative DEGs in root tip (**a**, **e**) and initialing crown roots (**c**). Probes used are antisense in **a, c** and **e**, sense in **b, d** and **f**. Bars = 200 μm.

Supplemental Table1. Primer used in this study.

Primers for ChIP analysis	Primer sequence
Chip 01g19694 F1	AGGCAACTCAATGAAAACTAACAAC
Chip 01g19694 R1	TAGAACCATCAGTAACCCAAGTTCA
Chip 01g46760 F1	GCTCCTCGGACCTTTCTTCC
Chip 01g46760 F2	GGGAGTAGTGGGTATCACCAATG
Chip 01g46760 R1	CCCTCACCAGAGAGAGCAAAA
Chip 01g46760 R2	TGTGAGGATTGCTATAGTTAAAGTTTATTTATG
Chip 01g54890 F	GCCAAACACAGCCTTAGAGGA
Chip 01g54890 R	GCGCTCTGTTTATTAATGCACG
Chip 02g57490 F1	TAGTAAGGCAGAGGACGTTGGC
Chip 02g57490 F2	AACCCACAACTAAACTACGGGC
Chip 02g57490 R1	GGAGGACAGGTAGGGTAGGGTG
Chip 02g57490 R2	AAATGTAGCCACTTTTTTTGCGA
Chip 05g06970 F1	TTACCCATTCTTCCATTAGCAGAAT
Chip 05g06970 F2	TTAGGCCCTGAACCTAGCAAATA
Chip 05g06970 R1	GATGCCCCATGCTTCGATAT
Chip 05g06970 R2	TGCTTGCTTAATTGACTTCCTACCT
Chip 08g35210 F1	ATTACTCAATTAGGTTACAATTATAGTGACAGG
Chip 08g35210 F2	GGCCTTCAGATCCAAGGCTT
Chip 08g35210 R1	AACCACTAGCAGATCACACCCA
Chip 08g35210 R2	TGATTAATGCTTGAAATTCAGAAAATGTAT
Chip 11g02130 F	GACCCTCGTAGACATATTTTGACCA
Chip 11g02130 R	ATGGCCTTGGAACCACCA
Chip 12g41680 F1	CTTTCTAGCAGCAAACACATTTAGC
Chip 12g41680 F2	ATCAGTTAAATGGTCTCCAGTGAAGC
Chip 12g41680 R1	TAATCATTTGGGATTTGACATTCAT
Chip 12g41680 R2	TCCGAGAATTGAAACTAACAAAGTTAAT
Primers for *in situ* hybridization	Primer sequence
01g19694 F	TACTTCGACACTGGCACAA
01g19694 R	GTCCTCGGCCTATCTAACA
03g10210 F	TCCTCCAGAACTGCCACC
03g10210 R	GCAAAGTCACCGCTAAAGAT
06g44930 F	CCGTGGGGTGATCCGTCT
06g44930 R	ACTGTGAACAGCGATTTTGC
07g38170 F	GGGATCAGTGCTGTGATGTG
07g38170 R	GAATGAAGTGAAGGAAGCCAC
12g41680 F	AGCAGCAGCCATTTCA
12g41680 R	GGTCCTGTGCAGAGTCATC
Primers for quantitative RT-PCR	Primer sequence
actin1 F	TGTATGCCAGTGGTCGTACCA
actin1 R	CCAGCAAGGTCGAGACGAA
rt-01g19694 F	CATCAACCAGAGGAAGAGGCA

rt-01g19694 R	GGCGGCATTGCTCAAAAAT
rt-01g46760 F	TTTGCAGCTGAGTTTGCTGG
rt-01g46760 R	AATAGCACACGCTCATCGGTC
rt-01g47070 F	GCCCCCAAGCCTGAGTAATTA
rt-01g47070 R	GATGCGTATGAACGGATCTCC
rt-01g54890 F	CGTACCTCCCGCTCAACGA
rt-01g54890 R	CGACGACGACGACGACGACA
rt-02g02400 F	AGGAGGCAGAAGGCGACGATACA
rt-02g02400 R	TCTTCACATGCTTGGCTTCACGTT
rt-02g33820 F	TGAACGCAAGAACGAACGG
rt-02g33820 R	AAAAGCGACGACGTATGACGA
rt-02g57490 F	GTGCAACGTCATAGTGAAGCAA
rt-02g57490 R	GGTCCAGTGATGTGTGATGTCA
rt-03g08754 F	CAATGCTTCTTACCCGCGA
rt-03g08754 R	GTTTCCAGCCATCACTTGGAG
rt-03g10210 F	CAAGTGGTTGCCATGGACTGA
rt-03g10210 R	GCAGAAGACGCCAAAACATGT
rt-03g11734 F	AATCCTCAGCGTTTTGCTGGT
rt-03g11734 R	TACGATCCCGATTCACGAAGC
rt-03g20910 F	TCCTAATGCAGAGCCTCCAAA
rt-03g20910 R	GCATGCCTAAAAATGTGGTGC
rt-03g29410 F	CCTCCTCAATGTCACCGATGA
rt-03g29410 R	TTTTCCACCATCCCTCTCACC
rt-03g46860 F	GCAAAACAGCAGAAATAGCGC
rt-03g46860 R	TCAATGGCAACATGCCTGA
rt-05g04490 F	GGCGAGCTTTGACAACCAGTA
rt-05g04490 R	TTGCCCATCTTCACCATGG
rt-05g06970 F	CCCAACACAACCGATGGAA
rt-05g06970 R	ACCTGTTGTCGAACTTGGTCG
rt-05g20020 F	GACCTCAGATGCCGATGATCTA
rt-05g20020 R	TCAGCTTTTAAGTGAGGCATGC
rt-06g11290 F	ATGGCACTTTCATCGCTGC
rt-06g11290 R	CCTGTTCAATGGTGCGTCAA
rt-06g44930 F	TGCCCCGACAAGTTTGGTTA
rt-06g44930 R	CGATGACATTTTCGCGCAG
rt-07g01410 F	TCGTGCTGCTCTTCTGATCGA
rt-07g01410 R	ACGACGAAACGACGTACGAAC
rt-07g05720 F	ACCCTTCAGTCCAATTCGCA
rt-07g05720 R	GGCTGAAGAGGAACGGAATGT
rt-07g34670 F	TCATTGGAGTAATGGCCCGTT
rt-07g34670 R	ATGCCTGAATTTGGTGGCG
rt-07g38170 F	GTGATGTGGTTGCTGTGTGGA
rt-07g38170 R	TGTTCTGTCTGTCCCTCCCAA
rt-07g48030 F	TGGTTTCATCAGCAAGTGCAC

rt-07g48030 F	TCCGTAATCCTCATGCCTTTG
rt-08g07180 F	AGTGACACAGATGAGAACCCGA
rt-08g07180 R	CAGTCCGGTCCCTATCATCTTT
rt-08g35210 F	TATGAAGAGCGTGATGCCAGG
rt-08g35210 R	CCTTCCAGTTAGGCCTTGCAA
rt-10g39680 F	GCGTCATCACCAACATCATCA
rt-10g39680 R	CATGTCGCAGTAGCGCTTGTA
rt-11g02130 F	GCCAGAGCACATTCTTTGCTG
rt-11g02130 F	CTTCAATGGCGGAGCTACTGA
rt-11g02130 R	TACTGGACATGCACACCGCTT
rt-11g02130 R	TGATGTCCCCCATCTTGATCA
rt-12g08130 F	GGAGCAGTAGAATCCTTGCAGG
rt-12g08130 R	CGCCAACCTCCATACAATCTG
rt-12g41680 F	CTGCCTGAATTTGGCAGCTT
rt-12g41680 R	AGCCATTTCAGATGGAGGAGG

Supplemental Table 2. RNA-seq reads and analysis data of *wox11* mutant and wild type (HY)

		Raw reads	clean reads	aligned concordantly exactly 1 time	aligned concordantly >1 times	Failed to align
wox11 mutant-1	R1	43807682	43552504 (99.42%)	24962963 (57.33%)	14364134 (32.99%)	4215871 (9.68%)
	R2	43807682	42196351 (96.32%)	24110420 (57.15%)	13869310 (32.88%)	4206741 (9.97%)
wox11 mutant-2	R1	48983384	48769075 (99.56%)	27772853 (56.96%)	16162117 (33.15%)	4824987 (9.90%)
	R2	48983384	47294787 (96.55%)	26846126 (56.78%)	15604675 (33.00%)	4833233 (10.22%)
HY-1	R1	48311766	48094639 (99.55%)	27131557 (56.41%)	16280434 (33.85%)	4681367 (9.73%)
	R2	48311766	46733899 (96.73%)	26126885 (55.91%)	15691740 (33.58%)	4912233 (10.51%)
HY-2	R1	53406529	53122962 (99.47%)	30245440 (56.94%)	17395082 (32.75%)	5475829 (10.31%)
	R2	53406529	51948938 (97.27%)	29474026 (56.74%)	17024232 (32.77%)	5446943 (10.49%)

Supplemental Table 3. WOX11 binding motif number in the down- or up-regulated genes

Down-regulated gene locus	promoter	gene-body	5`- UTR	exon	3`- UTR	intron
LOC_Os01g03330	1	0	0	0	0	0
LOC_Os01g07480	1	1	0	1	0	0
LOC_Os01g14550	1	0	0	0	0	0
LOC_Os01g19694	0	1	0	0	0	1
LOC_Os01g24420	0	2	0	1	0	1
LOC_Os01g24550	0	1	0	1	0	0
LOC_Os01g24560	0	1	0	0	1	0
LOC_Os01g24600	0	1	0	0	0	1
LOC_Os01g24700	1	1	0	0	0	1
LOC_Os01g36590	0	2	0	0	0	2
LOC_Os01g38229	0	7	0	0	0	7
LOC_Os01g43774	2	2	0	0	0	2
LOC_Os01g46760	1	2	0	0	0	2
LOC_Os01g47070	1	1	0	0	1	0
LOC_Os01g51860	0	1	0	1	0	0
LOC_Os01g53130	0	1	0	0	1	0
LOC_Os01g56420	1	0	0	0	0	0
LOC_Os01g58240	0	4	0	1	0	3
LOC_Os01g68720	1	0	0	0	0	0
LOC_Os01g69010	1	0	0	0	0	0
LOC_Os01g71820	0	1	0	0	1	0
LOC_Os01g72740	0	1	0	0	0	1

LOC_Os01g73840	1	2	0	1	0	1
LOC_Os01g74110	0	1	0	0	0	1
LOC_Os01g74340	1	0	0	0	0	0
LOC_Os02g01890	1	0	0	0	0	0
LOC_Os02g02400	1	1	0	0	0	1
LOC_Os02g04230	0	1	0	0	0	1
LOC_Os02g08490	0	4	0	2	0	2
LOC_Os02g09690	0	3	0	0	0	3
LOC_Os02g12080	0	1	0	0	1	0
LOC_Os02g12350	0	2	0	1	0	1
LOC_Os02g29150	0	1	0	0	0	1
LOC_Os02g33820	1	0	0	0	0	0
LOC_Os02g34190	1	0	0	0	0	0
LOC_Os02g36590	1	2	0	0	1	1
LOC_Os02g36595	1	1	0	0	0	1
LOC_Os02g38392	0	5	0	1	0	4
LOC_Os02g44200	1	0	0	0	0	0
LOC_Os02g51680	0	1	0	0	0	1
LOC_Os02g52340	0	1	0	0	0	1
LOC_Os02g52930	0	1	0	0	0	1
LOC_Os02g54140	2	0	0	0	0	0
LOC_Os02g54180	1	0	0	0	0	0
LOC_Os02g57490	1	1	0	0	1	0
LOC_Os03g03034	0	3	0	0	0	3
LOC_Os03g04070	0	1	0	0	0	1
LOC_Os03g05334	1	0	0	0	0	0

LOC_Os03g08754	0	3	0	0	0	3
LOC_Os03g10210	0	1	0	0	0	1
LOC_Os03g10640	0	3	0	0	1	2
LOC_Os03g11734	0	6	0	0	0	6
LOC_Os03g12950	0	2	1	0	1	0
LOC_Os03g15370	1	0	0	0	0	0
LOC_Os03g15890	0	1	0	0	0	1
LOC_Os03g16920	0	1	0	0	1	0
LOC_Os03g18850	3	0	0	0	0	0
LOC_Os03g20120	1	0	0	0	0	0
LOC_Os03g20420	0	2	0	0	0	2
LOC_Os03g20910	0	1	0	1	0	0
LOC_Os03g22590	1	5	0	3	0	2
LOC_Os03g25030	1	1	0	0	0	1
LOC_Os03g28300	0	2	0	1	0	1
LOC_Os03g29410	4	1	0	0	0	1
LOC_Os03g41170	0	1	0	0	0	1
LOC_Os03g45970	0	2	0	0	0	2
LOC_Os03g46860	0	3	0	1	0	2
LOC_Os03g47860	1	0	0	0	0	0
LOC_Os03g55050	0	1	0	1	0	0
LOC_Os03g55080	1	1	0	0	0	1
LOC_Os03g58190	1	1	0	1	0	0
LOC_Os03g64100	1	0	0	0	0	0
LOC_Os04g01690	1	1	0	0	0	1
LOC_Os04g20200	0	1	0	0	0	1

LOC_Os04g28210	0	1	0	0	0	1
LOC_Os04g36740	0	1	0	1	0	0
LOC_Os04g40990	0	1	0	1	0	0
LOC_Os04g57130	0	1	1	0	0	0
LOC_Os04g59020	0	2	0	0	2	0
LOC_Os05g04490	1	0	0	0	0	0
LOC_Os05g06970	1	0	0	0	0	0
LOC_Os05g07710	0	1	0	0	0	1
LOC_Os05g08750	2	1	1	0	0	0
LOC_Os05g10370	1	0	0	0	0	0
LOC_Os05g11428	0	3	0	1	0	2
LOC_Os05g20020	0	1	0	0	1	0
LOC_Os05g33644	1	1	0	0	0	1
LOC_Os05g33700	1	0	0	0	0	0
LOC_Os05g33710	0	2	0	0	0	2
LOC_Os05g37880	0	2	0	1	1	0
LOC_Os05g38120	0	3	0	0	0	3
LOC_Os05g38270	0	2	0	1	0	1
LOC_Os05g47690	1	1	0	0	0	1
LOC_Os05g48570	1	0	0	0	0	0
LOC_Os06g02210	0	2	0	1	1	0
LOC_Os06g07200	0	1	0	0	0	1
LOC_Os06g07300	0	1	0	0	0	1
LOC_Os06g22150	2	0	0	0	0	0
LOC_Os06g33100	0	1	0	0	1	0
LOC_Os06g38750	0	1	0	0	0	1

LOC_Os06g43860	1	1	0	0	0	1
LOC_Os06g44610	2	1	0	0	0	1
LOC_Os06g44930	1	0	0	0	0	0
LOC_Os06g46340	0	2	0	0	0	2
LOC_Os06g46350	1	0	0	0	0	0
LOC_Os06g50830	1	0	0	0	0	0
LOC_Os07g01410	0	1	0	0	0	1
LOC_Os07g03910	1	0	0	0	0	0
LOC_Os07g04450	0	2	0	0	0	2
LOC_Os07g05720	0	1	0	0	0	1
LOC_Os07g08000	0	1	0	1	0	0
LOC_Os07g23430	0	2	0	0	2	0
LOC_Os07g28890	1	0	0	0	0	0
LOC_Os07g29600	1	3	1	0	0	2
LOC_Os07g34670	1	0	0	0	0	0
LOC_Os07g38170	1	0	0	0	0	0
LOC_Os07g39070	0	3	0	0	0	3
LOC_Os07g39210	0	1	0	1	0	0
LOC_Os07g40240	1	1	1	0	0	0
LOC_Os07g42160	1	0	0	0	0	0
LOC_Os07g48020	0	1	0	1	0	0
LOC_Os07g48030	1	1	0	1	0	0
LOC_Os08g01030	0	2	0	1	0	1
LOC_Os08g01510	1	0	0	0	0	0
LOC_Os08g02200	2	0	0	0	0	0
LOC_Os08g07180	1	0	0	0	0	0

LOC_Os08g09300	0	1	0	0	1	0
LOC_Os08g09690	0	3	0	1	0	2
LOC_Os08g14860	0	2	0	0	0	2
LOC_Os08g34280	1	2	0	0	0	2
LOC_Os08g35210	0	3	0	0	1	2
LOC_Os08g35948	0	2	0	1	0	1
LOC_Os08g36860	0	4	0	1	0	3
LOC_Os08g40680	1	0	0	0	0	0
LOC_Os08g40940	0	2	0	1	0	1
LOC_Os08g41880	1	0	0	0	0	0
LOC_Os08g44180	0	1	0	0	0	1
LOC_Os09g01290	0	1	0	0	0	1
LOC_Os09g03630	0	1	0	0	0	1
LOC_Os09g04995	0	1	1	0	0	0
LOC_Os09g07440	0	3	0	0	0	3
LOC_Os09g07450	0	2	0	0	0	2
LOC_Os09g09570	0	1	0	1	0	0
LOC_Os09g13650	1	0	0	0	0	0
LOC_Os09g18010	0	3	0	0	0	3
LOC_Os09g23140	1	1	0	1	0	0
LOC_Os09g23150	0	1	0	0	0	1
LOC_Os09g24490	0	1	0	1	0	0
LOC_Os09g26500	0	1	0	0	0	1
LOC_Os09g31482	1	0	0	0	0	0
LOC_Os09g33740	0	1	0	0	0	1
LOC_Os09g35860	1	0	0	0	0	0

LOC_Os10g02300	0	2	0	1	1	0
LOC_Os10g18820	0	1	0	1	0	0
LOC_Os10g28020	2	1	0	0	0	1
LOC_Os10g28030	1	1	0	0	0	1
LOC_Os10g29650	0	1	0	1	0	0
LOC_Os10g39640	0	1	0	0	1	0
LOC_Os10g39680	1	0	0	0	0	0
LOC_Os10g41060	0	2	0	0	2	0
LOC_Os11g02130	1	0	0	0	0	0
LOC_Os11g02570	1	1	0	1	0	0
LOC_Os11g04409	0	1	1	0	0	0
LOC_Os11g05800	2	0	0	0	0	0
LOC_Os11g14570	0	1	0	0	1	0
LOC_Os11g17504	0	1	0	1	0	0
LOC_Os11g23930	1	0	0	0	0	0
LOC_Os11g31050	0	2	0	1	0	1
LOC_Os11g40590	1	4	1	1	0	2
LOC_Os12g04340	1	0	0	0	0	0
LOC_Os12g07310	0	1	0	0	1	0
LOC_Os12g08090	0	1	0	0	0	1
LOC_Os12g08130	0	1	0	1	0	0
LOC_Os12g08760	0	4	0	0	0	4
LOC_Os12g09540	0	1	0	0	0	1
LOC_Os12g12590	0	1	0	0	0	1
LOC_Os12g12600	1	3	0	0	0	3
LOC_Os12g13720	0	5	0	1	0	4

LOC_Os12g13730	1	0	0	0	0	0
LOC_Os12g28590	1	0	0	0	0	0
LOC_Os12g36240	0	1	0	1	0	0
LOC_Os12g39220	1	0	0	0	0	0
LOC_Os12g41680	0	3	0	0	0	3
LOC_Os12g42810	0	1	0	1	0	0

Up-regulated gene locus	promoter	gene-body	5`- UTR	exon	3`- UTR	intron
LOC_Os11g31770	0	5	0	0	0	5
LOC_Os11g24374	0	5	0	1	0	4
LOC_Os06g16790	0	4	0	2	0	2
LOC_Os09g25860	0	4	0	0	0	4
LOC_Os02g58740	2	2	2	0	0	0
LOC_Os12g10340	1	2	0	2	0	0
LOC_Os03g22010	0	3	0	1	0	2
LOC_Os03g21310	0	3	0	0	0	3
LOC_Os11g34460	0	3	0	3	0	0
LOC_Os04g38430	1	2	1	0	0	1
LOC_Os02g05560	0	3	0	1	0	2
LOC_Os01g64430	1	2	1	0	1	0
LOC_Os03g09970	1	2	1	0	0	1
LOC_Os04g02780	1	2	0	0	0	2
LOC_Os01g15740	0	3	0	0	0	3
LOC_Os05g26660	0	3	0	2	0	1
LOC_Os03g53660	0	3	0	0	0	3
LOC_Os05g39790	0	2	0	2	0	0

LOC_Os12g38590	0	2	0	0	0	2
LOC_Os03g53590	1	1	0	0	0	1
LOC_Os07g03040	0	2	0	0	0	2
LOC_Os10g40460	1	1	0	0	1	0
LOC_Os01g37630	1	1	0	1	0	0
LOC_Os04g55280	2	0	0	0	0	0
LOC_Os04g04790	0	2	0	0	0	2
LOC_Os04g04800	2	0	0	0	0	0
LOC_Os09g37060	0	2	0	2	0	0
LOC_Os01g22450	1	1	0	0	0	1
LOC_Os12g39710	0	2	0	0	2	0
LOC_Os11g43700	1	1	0	1	0	0
LOC_Os12g17480	1	1	0	0	0	1
LOC_Os01g49950	0	2	0	0	0	2
LOC_Os04g39160	1	1	0	1	0	0
LOC_Os07g13660	1	1	0	0	0	1
LOC_Os01g62200	1	1	0	1	0	0
LOC_Os01g16310	0	1	0	0	0	1
LOC_Os04g38010	1	0	0	0	0	0
LOC_Os02g27580	0	1	0	0	0	1
LOC_Os07g32680	1	0	0	0	0	0
LOC_Os10g07160	0	1	0	0	0	1
LOC_Os10g37340	1	0	0	0	0	0
LOC_Os01g61044	0	1	0	0	0	1
LOC_Os10g41330	1	0	0	0	0	0
LOC_Os02g21810	1	0	0	0	0	0

LOC_Os09g37310	0	1	0	1	0	0
LOC_Os01g35950	1	0	0	0	0	0
LOC_Os04g23910	0	1	0	0	0	1
LOC_Os05g30570	1	0	0	0	0	0
LOC_Os10g38720	0	1	0	1	0	0
LOC_Os06g11290	0	1	0	0	1	0
LOC_Os01g54890	1	0	0	0	0	0
LOC_Os03g44740	1	0	0	0	0	0
LOC_Os03g05910	0	1	0	0	0	1
LOC_Os10g31740	0	1	0	0	0	1
LOC_Os06g29470	1	0	0	0	0	0
LOC_Os03g43970	1	0	0	0	0	0
LOC_Os05g34830	0	1	0	0	0	1
LOC_Os09g38130	0	1	0	0	0	1
LOC_Os01g41430	0	1	0	0	1	0
LOC_Os01g50570	0	1	0	0	0	1
LOC_Os01g57030	0	1	0	0	1	0
LOC_Os09g23820	1	0	0	0	0	0
LOC_Os07g44860	1	0	0	0	0	0
LOC_Os05g42350	1	0	0	0	0	0
LOC_Os03g37570	0	1	0	1	0	0
LOC_Os09g34250	0	1	0	0	1	0
LOC_Os01g47130	0	1	0	1	0	0
LOC_Os01g14770	0	1	0	0	0	1
LOC_Os01g70540	0	1	0	0	0	1
LOC_Os08g18920	0	1	0	1	0	0

LOC_Os05g40910	0	1	0	0	1	0
LOC_Os01g50030	0	1	0	1	0	0
LOC_Os01g50032	0	1	0	1	0	0
LOC_Os01g53030	1	0	0	0	0	0
LOC_Os04g14810	1	0	0	0	0	0
LOC_Os10g31530	1	0	0	0	0	0
LOC_Os04g46970	0	1	1	0	0	0
LOC_Os05g44630	0	1	0	1	0	0
LOC_Os04g57410	0	1	0	0	0	1
LOC_Os03g08114	0	1	0	1	0	0
LOC_Os06g17020	1	0	0	0	0	0
LOC_Os07g33270	1	0	0	0	0	0
LOC_Os08g14190	0	1	0	0	0	1
LOC_Os08g08960	1	0	0	0	0	0
LOC_Os08g27020	1	0	0	0	0	0
LOC_Os10g27010	0	1	0	0	0	1
LOC_Os10g42900	0	1	0	0	1	0
LOC_Os12g07560	1	0	0	0	0	0
LOC_Os01g50520	0	1	0	0	0	1
LOC_Os12g24390	1	0	0	0	0	0
LOC_Os01g61080	0	1	0	0	0	1
LOC_Os01g64840	1	0	0	0	0	0
LOC_Os02g41904	0	1	0	0	1	0

Supplemental Table 4. Root-specific differentially expressed genes in *wox11* root tips

Down-regulated gene locus	With motif or not	Reported name	annotation	Fold Change
LOC_Os09g24370	N		expressed protein	0.342509997
LOC_Os07g01410	Y		peroxidase precursor, putative, expressed	0.185568988
LOC_Os01g24600	Y		fruit bromelain precursor, putative, expressed	0.35921611
LOC_Os01g24550	Y		fruit bromelain precursor, putative, expressed	0.385998356
LOC_Os11g20160	N		O-methyltransferase, putative, expressed	0.499071297
LOC_Os01g68720	Y		keratin, type I cytoskeletal 9, putative, expressed	0.489274347
LOC_Os10g18820	Y		dirigent, putative, expressed	0.154301735
LOC_Os12g38010	N		metallothionein, putative, expressed	0.493673353
LOC_Os01g24560	Y		fruit bromelain precursor, putative, expressed	0.2514304
LOC_Os01g68730	N		RNA-binding protein FUS, putative, expressed	0.400912353
LOC_Os05g06970	Y		peroxidase precursor, putative, expressed	0.294982852
LOC_Os10g18760	N		dirigent, putative, expressed	0.270145936
LOC_Os03g25030	Y		GDSL-like lipase/acylhydrolase, putative, expressed	0.400225021
LOC_Os06g32160	N		THION7 - Plant thionin family protein precursor, expressed	0.063332729
LOC_Os01g71380	N		glycosyl hydrolases family 17, putative, expressed	0.257565243
LOC_Os07g23430	Y		fatty acid desaturase, putative, expressed	0.385215317
LOC_Os06g32990	N		peroxidase precursor, putative, expressed	0.488877792
LOC_Os11g14910	N		NADP-dependent oxidoreductase, putative, expressed	0.248550623
LOC_Os02g17900	N		glycosyl hydrolases family 16, putative, expressed	0.47525169
LOC_Os08g37250	N	OspPLAIIζ	patatin, putative, expressed	0.49281716
LOC_Os01g51570	N		glycosyl hydrolases family 17, putative, expressed	0.472271566
LOC_Os12g36240	Y		inhibitor I family protein, putative, expressed	0.395449071

LOC_Os05g48740	N		expressed protein	0.363803983
LOC_Os03g13160	N		non-symbiotic hemoglobin 2, putative, expressed	0.390754622
LOC_Os03g20210	N		aspartic proteinase nepenthesin precursor, putative, expressed	0.450749353
LOC_Os10g39300	N		aspartic proteinase nepenthesin, putative, expressed	0.300368218
LOC_Os03g25330	N		peroxidase precursor, putative, expressed	0.446238292
LOC_Os03g28300	Y		protein kinase domain containing protein, expressed	0.389472503
LOC_Os01g60930	N		expressed protein	0.35206618
LOC_Os01g15340	N	OsRAA1	flowering promoting factor-like 1, putative, expressed	0.413244827
LOC_Os08g24200	N		expressed protein	0.398815702
LOC_Os03g52860	N	L-2	lipoxygenase, putative, expressed	0.48197727
LOC_Os10g04800	N	crl-2	expressed protein	0.419649385
LOC_Os07g35560	N		glucan endo-1,3-beta-glucosidase precursor, putative, expressed	0.205873564
LOC_Os10g02300	Y	OsPCR1	uncharacterized Cys-rich domain containing protein, putative, expressed	0.260474533
LOC_Os01g72910	N	Asr2;ASR4 ;OsASR6	abscisic stress-ripening, putative, expressed	0.263294913
LOC_Os11g23930	Y		expressed protein	0.375912665
LOC_Os08g36040	N		plant viral response family protein, putative, expressed	0.489243918
LOC_Os03g20420	Y		alpha-N-arabinofuranosidase A, putative, expressed	0.315020213
LOC_Os12g12600	Y		dirigent, putative, expressed	0.125657234
LOC_Os04g56420	N		retrotransposon protein, putative, unclassified, expressed	0.377045304
LOC_Os05g04490	Y		peroxidase precursor, putative, expressed	0.105474955
LOC_Os11g44430	N		protein kinase, putative, expressed	0.486694562
LOC_Os08g07690	N		expressed protein	0.358977203
LOC_Os09g31410	N		Os9bglu29 - beta-glucosidase homologue, similar to Os4bglu12 exoglucanase, expressed	0.228295532
LOC_Os03g55050	Y		UDP-glucoronosyl and UDP-glucosyl transferase domain containing protein,	0.491658655

			expressed	
LOC_Os03g15080	N		expressed protein	0.299945638
LOC_Os01g41240	N		hydrolase, alpha/beta fold family domain containing protein, expressed	0.150749123
LOC_Os08g40940	Y		expressed protein	0.468252822
LOC_Os06g16300	N		BRASSINOSTEROID INSENSITIVE 1-associated receptor kinase 1 precursor, putative, expressed	0.437705512
LOC_Os02g12080	Y		expressed protein	0.343501403
LOC_Os08g24300	N		expressed protein	0.139902935
LOC_Os06g21820	N		jasmonate O-methyltransferase, putative, expressed	0.434170284
LOC_Os11g47460	N		MYB family transcription factor, putative, expressed	0.475415759
LOC_Os01g74110	Y	OsZIP1	metal cation transporter, putative, expressed	0.282580373
LOC_Os11g05800	Y		HVA22, putative, expressed	0.396845485
LOC_Os03g13210	N		peroxidase precursor, putative, expressed	0.305254715
LOC_Os01g45140	N		anthocyanin 3-O-beta-glucosyltransferase, putative, expressed	0.48896993
LOC_Os11g12530	N		receptor-like protein kinase 5 precursor, putative, expressed	0.471540762
LOC_Os02g55890	N	OVP3	inorganic H+ pyrophosphatase, putative, expressed	0.430109423
LOC_Os03g58980	N		Cupin domain containing protein, expressed	0.285676865
LOC_Os12g03740	N		OsFBX438 - F-box domain containing protein, expressed	0.258212005
LOC_Os08g40720	N		FAD-binding and arabino-lactone oxidase domains containing protein, putative, expressed	0.083700058
LOC_Os12g12590	Y		NADP-dependent oxidoreductase, putative, expressed	0.277788595
LOC_Os06g21690	N		expressed protein	0.355436571
LOC_Os04g34600	N	Asr4; ASR6; OsASR2	abscisic stress-ripening, putative, expressed	0.321127399
LOC_Os04g53860	N		reductase, putative, expressed	0.36969427

LOC_Os07g48020	Y		peroxidase precursor, putative, expressed	0.401572019
LOC_Os10g34040	N		nodulin, putative, expressed	0.224737672
LOC_Os03g29410	Y		tyrosine protein kinase domain containing protein, putative, expressed	0.495354851
LOC_Os12g28590	Y		ATPase 2, putative, expressed	0.261805397
LOC_Os02g13510	N		receptor-like protein kinase 5 precursor, putative, expressed	0.499087334
LOC_Os01g72740	Y		cytochrome P450, putative, expressed	0.351798543
LOC_Os09g31210	N		AGC_PVPK_like_CDK8.1 - ACG kinases include homologs to PKA, PKG and PKC, expressed	0.491198638
LOC_Os06g33100	Y		peroxidase precursor, putative, expressed	0.334132017
LOC_Os08g08980	N	OsGLP8-4; GER1	cupin domain containing protein, expressed	0.424215963
LOC_Os06g02210	Y		bark storage protein A precursor, putative, expressed	0.406119258
LOC_Os06g03810	N		expressed protein	0.224382273
LOC_Os12g09540	Y		phosphoribosylamine--glycine ligase, putative, expressed	0.476745156
LOC_Os01g71710	N		amino acid permease family protein, putative, expressed	0.307302616
LOC_Os01g56420	Y	COPT1	ctr copper transporter family protein, putative, expressed	0.369454023
LOC_Os09g23140	Y		endonuclease/exonuclease/phosphatase family domain containing protein, expressed	0.443954728
LOC_Os01g38229	Y		peptidyl-prolyl isomerase, putative, expressed	0.385982278
LOC_Os01g47070	Y		glycosyl hydrolase, putative, expressed	0.301164472
LOC_Os01g74410	N	OsMYB48	MYB family transcription factor, putative, expressed	0.355838371
LOC_Os03g13520	N		endonuclease/exonuclease/phosphatase family domain containing protein, expressed	0.367021277
LOC_Os12g07310	Y		citrate-binding protein precursor, putative, expressed	0.172638022
LOC_Os02g11770	N		expressed protein	0.423049409
LOC_Os02g54180	Y		expressed protein	0.478094933

LOC_Os06g38750	Y		proline-rich family protein, putative, expressed	0.122927675
LOC_Os08g40380	N		sulfotransferase domain containing protein, expressed	0.27043508
LOC_Os03g18850	Y	JIOsPR10	pathogenesis-related Bet v I family protein, putative, expressed	0.420236206
LOC_Os06g41740	N		transposon protein, putative, CACTA, En/Spm sub-class, expressed	0.418029915
LOC_Os06g49660	N		transferase family protein, putative, expressed	0.417976369
LOC_Os01g71700	N		amino acid permease family protein, putative, expressed	0.344595805
LOC_Os07g09310	N		thioredoxin, putative, expressed	0.417586805
LOC_Os02g57490	Y		DUF260 domain containing protein, putative, expressed	0.432726583
LOC_Os06g36070	N		expressed protein	0.404300445
LOC_Os03g44100	N		phosphonate metabolism protein-related, putative, expressed	0.356672548
LOC_Os12g39220	Y		ZOS12-08 - C2H2 zinc finger protein, expressed	0.475143816
LOC_Os06g07300	Y		jacalin-like lectin domain containing protein, expressed	0.269973319
LOC_Os04g57130	Y		dirigent-like protein pDIR17, putative, expressed	0.261671233
LOC_Os07g44370	N		dirigent, putative, expressed	0.269497336
LOC_Os03g09980	N		sulfate transporter, putative, expressed	0.407744659
LOC_Os01g09700	N	OsACS5	aminotransferase, classes I and II, domain containing protein, expressed	0.40881234
LOC_Os04g01690	Y		pyridoxal-dependent decarboxylase protein, putative, expressed	0.193768113
LOC_Os03g12150	N		serine/threonine-protein kinase receptor precursor, putative, expressed	0.27183457
LOC_Os02g01890	Y		cytochrome P450, putative, expressed	0.413000515
LOC_Os02g52930	Y		integral membrane protein DUF6 containing protein, expressed	0.44099295
LOC_Os02g33820	Y	Asr3; OsASR3; ASR1	abscisic stress-ripening, putative, expressed	0.318188813
LOC_Os06g07200	Y		syntaxin, putative, expressed	0.394200459
LOC_Os12g36850	N		pathogenesis-related Bet v I family protein, putative, expressed	0.192285712
LOC_Os02g53750	N		tyrosine protein kinase domain containing protein, putative, expressed	0.411225512

LOC_Os03g05334	Y		expressed protein	0.36417202
LOC_Os11g05740	N		B3 DNA binding domain containing protein, expressed	0.314242907
LOC_Os06g41930	N		zinc-binding protein, putative, expressed	0.433060016
LOC_Os08g01480	N		cytochrome P450, putative, expressed	0.360414723
LOC_Os10g40490	N		U-box domain containing protein, expressed	0.459001311
LOC_Os04g56390	N		expressed protein	0.267047598
LOC_Os03g11734	Y	OsFRDL1	MATE efflux protein, putative, expressed	0.308831076
LOC_Os04g28210	Y		verticillium wilt disease resistance protein, putative, expressed	0.493070263
LOC_Os09g29820	N	OsbZIP73	bZIP transcription factor domain containing protein, expressed	0.462192722
LOC_Os07g34070	N		auxin-induced protein 5NG4, putative, expressed	0.242452648
LOC_Os07g36544	N		serine/threonine-protein kinase receptor precursor, putative, expressed	0.446369489
LOC_Os12g13730	Y		pleiotropic drug resistance protein 2, putative, expressed	0.453994592
LOC_Os01g43460	N		C4-dicarboxylate transporter/malic acid transport protein, expressed	0.236786738
LOC_Os06g09620	N		expressed protein	0.353845871
LOC_Os03g08754	Y	OsMDP1	OsMADS47 - MADS-box family gene with MIKCc type-box, expressed	0.070232782
LOC_Os09g26960	N		cytochrome P450, putative, expressed	0.361678489
LOC_Os01g38380	N		plant protein of unknown function domain containing protein, expressed	0.49366994
LOC_Os02g02400	Y	OsCATA	catalase isozyme A, putative, expressed	0.439709495
LOC_Os03g10640	Y		calcium-transporting ATPase, plasma membrane-type, putative, expressed	0.496519191
LOC_Os01g58240	Y		OsSub6 - Putative Subtilisin homologue, expressed	0.468338229
LOC_Os07g48030	Y		peroxidase precursor, putative, expressed	0.262527383
LOC_Os05g50470	N		expressed protein	0.467422952
LOC_Os02g52180	N		plastocyanin-like domain containing protein, putative, expressed	0.47945222
LOC_Os09g22510	N		expressed protein	0.435779073
LOC_Os10g41060	Y		expressed protein	0.464558438
LOC_Os05g10370	Y		acid phosphatase, putative, expressed	0.42918221

LOC_Os05g48210	N		expressed protein	0.427176644
LOC_Os09g23780	N		expressed protein	0.360622131
LOC_Os11g15250	N		THION31 - Plant thionin family protein precursor, expressed	0.269894973
LOC_Os04g56030	N		glycine-rich cell wall structural protein precursor, putative, expressed	0.313196962
LOC_Os01g03330	Y		BBTI3 - Bowman-Birk type bran trypsin inhibitor precursor, expressed	0.45124553
LOC_Os12g04340	Y		sulfotransferase domain containing protein, expressed	0.335138828
LOC_Os03g61490	N		expressed protein	0.244762899
LOC_Os08g19650	N		homeobox protein knotted-1, putative, expressed	0.473045584
LOC_Os05g50580	N		OsSCP30 - Putative Serine Carboxypeptidase homologue, expressed	0.327392377
LOC_Os03g36080	N		expressed protein	0.420856574
LOC_Os05g51420	N		hypersensitive-induced response protein, putative, expressed	0.482857749
LOC_Os05g33644	Y		inosine-uridine preferring nucleoside hydrolase family protein, putative, expressed	0.414888212
LOC_Os02g41460	N		DUF640 domain containing protein, putative, expressed	0.455300233
LOC_Os01g43530	N		late embryogenesis abundant protein, putative, expressed	0.380156662
LOC_Os02g43170	N		B-box zinc finger family protein, putative, expressed	0.158553428
LOC_Os08g26850	N		plant protein of unknown function domain containing protein, expressed	0.484978967
LOC_Os08g02200	Y		expressed protein	0.346240883
LOC_Os04g47810	N		ethylene-responsive protein related, putative, expressed	0.398608597
LOC_Os09g18010	Y		expressed protein	0.451720863
LOC_Os04g37490	N		oxidoreductase, aldo/keto reductase family protein, putative, expressed	0.269339599
LOC_Os12g37970	N		MYB family transcription factor, putative, expressed	0.405831919
LOC_Os08g41880	Y		nucleotide pyrophosphatase/phosphodiesterase, putative, expressed	0.318152696
LOC_Os12g13720	Y		Plant PDR ABC transporter associated domain containing protein, expressed	0.453994592
LOC_Os04g35660	N		no apical meristem protein, putative, expressed	0.47111153
LOC_Os03g08320	N	OsJAZ11;	ZIM domain containing protein, putative, expressed	0.498108488

		OsTIFY11c		
LOC_Os01g14550	Y		pathogen-related protein, putative, expressed	0.465559695
LOC_Os05g05030	N		expressed protein	0.369321827
LOC_Os06g05020	N		early nodulin 93 ENOD93 protein, putative, expressed	0.195117129
LOC_Os12g28100	N		NBS-LRR disease resistance protein, putative, expressed	0.43612464
LOC_Os03g46860	Y		helix-loop-helix DNA-binding protein, putative, expressed	0.275893404
LOC_Os04g36740	Y		potassium channel SKOR, putative, expressed	0.298006609
LOC_Os10g39680	Y		CHIT14 - Chitinase family protein precursor, expressed	0.357746662
LOC_Os07g38170	Y		remorin, putative, expressed	0.457897977
LOC_Os09g35860	Y		ras-related protein, putative, expressed	0.456420855
LOC_Os06g47640	N	OsCML29	OsCML29 - Calmodulin-related calcium sensor protein, expressed	0.260457082
LOC_Os12g08760	Y		carboxyvinyl-carboxyphosphonate phosphorylmutase, putative, expressed	0.340481014
LOC_Os02g36590	Y		CPuORF19 - conserved peptide uORF-containing transcript, expressed	0.436599855
LOC_Os04g45480	N		heat shock protein STI, putative, expressed	0.49564746
LOC_Os11g04409	Y		expressed protein	0.434444157
LOC_Os03g12860	N		homeobox associated leucine zipper, putative, expressed	0.325995558
LOC_Os06g13210	N		peptide transporter PTR2, putative, expressed	0.308205262
LOC_Os11g02570	Y		esterase, putative, expressed	0.413320303
LOC_Os11g02130	Y		peroxidase precursor, putative, expressed	0.463159657
LOC_Os02g12350	Y		histone deacetylase, putative, expressed	0.497801011
LOC_Os08g39150	N		expressed protein	0.489791117
LOC_Os08g08084	N		expressed protein	0.249112186
LOC_Os07g03910	Y		transporter, major facilitator superfamily domain containing protein, expressed	0.478333439
LOC_Os06g43860	Y		homeobox protein knotted-1, putative, expressed	0.468521287
LOC_Os04g47310	N		expressed protein	0.407047755
LOC_Os11g35080	N		expressed protein	0.374754222

LOC_Os05g08554	N		expressed protein	0.40804297
LOC_Os04g10750	N	OsPht1;4; OsPT4	inorganic phosphate transporter, putative, expressed	0.45276046
LOC_Os03g15370	Y	UbL402	ubiquitin fusion protein, putative, expressed	0.498563792
LOC_Os01g64470	N		harpin-induced protein 1 domain containing protein, expressed	0.340824953
LOC_Os08g01030	Y	OsZIP16	metal cation transporter, putative, expressed	2.774297918
LOC_Os04g59020	Y		integral membrane protein, putative, expressed	0.475400405
LOC_Os05g08900	N		expressed protein	0.318599439
LOC_Os09g35790	N	OsHsfB2c	HSF-type DNA-binding domain containing protein, expressed	0.338428351
LOC_Os04g09390	N		HEV3 - Hevein family protein precursor, expressed	0.397567114
LOC_Os07g28890	Y		ethylene-responsive protein related, putative, expressed	0.487047357

UP-regulated gene locus	with motif or not	Reported name	annotation	Fold Change
LOC_Os10g31530	N		glycine-rich cell wall protein, putative, expressed	2.268908602
LOC_Os04g11040	N		expressed protein	2.489487112
LOC_Os04g46810	N		LTPL120 - Protease inhibitor/seed storage/LTP family protein precursor, expressed	2.091987009
LOC_Os06g16790	N		go35 NBS-LRR, putative, expressed	6.819676027
LOC_Os12g24390	N		retrotransposon protein, putative, unclassified, expressed	2.053368983
LOC_Os01g66900	N		expressed protein	3.736083498
LOC_Os01g64840	N		aspartic proteinase nepenthesin-1 precursor, putative, expressed	2.01725282
LOC_Os10g31560	N		glycine-rich cell wall structural protein 2 precursor, putative, expressed	3.734848164
LOC_Os04g23910	N	OsMADS25	OsMADS25 - MADS-box family gene with MIKCc type-box, expressed	3.414605878
LOC_Os10g40480	N		LTPL143 - Protease inhibitor/seed storage/LTP family protein precursor, expressed	4.802608759

LOC_Os01g50520	N		cytochrome P450 monooxygenase CYP711A12, putative, expressed	2.067258785
LOC_Os10g03390	N		transferase family protein, putative, expressed	4.711394091
LOC_Os12g24330	N		expressed protein	2.431125279
LOC_Os03g02939	N		peroxidase precursor, putative, expressed	2.040954183
LOC_Os08g04350	N		plastocyanin-like domain containing protein, putative, expressed	2.456104207
LOC_Os01g50580	N	SLB2	cytochrome P450, putative, expressed	2.603583291
LOC_Os04g38430	N		nodulin, putative, expressed	2.685580155
LOC_Os08g01950	N		transferase family protein, putative, expressed	2.002874378
LOC_Os01g03890	N		DUF260 domain containing protein, putative, expressed	2.673601202
LOC_Os03g44170	N		glutathione S-transferase, putative, expressed	6.333681991
LOC_Os03g09970	N		sulfate transporter, putative, expressed	2.116968901
LOC_Os10g40470	N		LTPL142 - Protease inhibitor/seed storage/LTP family protein precursor, putative, expressed	7.866752047
LOC_Os06g08120	N		plant protein of unknown function domain containing protein, expressed	2.476467038
LOC_Os10g38720	N		glutathione S-transferase, putative, expressed	3.137664084
LOC_Os06g17020	N		anthocyanin 3-O-beta-glucosyltransferase, putative, expressed	2.191963274
LOC_Os02g43410	N	OsYSL15	transposon protein, putative, unclassified, expressed	2.931416641
LOC_Os09g20220	N		glutathione S-transferase, putative, expressed	2.515866858
LOC_Os07g48060	N		peroxidase precursor, putative, expressed	9.229173817
LOC_Os10g31780	N		oxidoreductase, short chain dehydrogenase/reductase family domain containing protein, expressed	4.147933645
LOC_Os03g07510	N	PGL1	expressed protein	2.523217775
LOC_Os08g13980	N		glycosyl hydrolases family 16, putative, expressed	2.793279961
LOC_Os06g21950	N	OsPht1;10; OsPT10	inorganic phosphate transporter, putative, expressed	2.615991719
LOC_Os12g10340	N		NBS-LRR type resistance protein, putative, expressed	12.37034996

LOC_Os11g41850	N		transporter family protein, putative, expressed	2.50620903
LOC_Os11g10290	N		S-domain receptor-like protein kinase, putative, expressed	2.087155702
LOC_Os06g29470	N		peroxidase precursor, putative, expressed	2.836297865
LOC_Os05g13900	N	RePRP1.2	retrotransposon protein, putative, unclassified, expressed	2.859626047
LOC_Os04g52780	N	OsFLS2	leucine-rich repeat receptor protein kinase EXS precursor, putative, expressed	2.155335351
LOC_Os01g41430	N		UDP-glucoronosyl and UDP-glucosyl transferase, putative, expressed	2.605496678
LOC_Os02g33780	N		serine-aspartate repeat-containing protein I precursor, putative, expressed	2.095951734
LOC_Os02g39330	N		CHIT1 - Chitinase family protein precursor, expressed	2.194432709
LOC_Os01g20950	N		sulfotransferase domain containing protein, expressed	3.631818807
LOC_Os10g36980	N		transposon protein, putative, unclassified, expressed	2.064299512
LOC_Os02g42640	N		CBS domain-containing protein, putative, expressed	2.813497554
LOC_Os07g38840	N		expressed protein	2.323954698
LOC_Os01g54890	N	OsERF922	ethylene-responsive transcription factor 2, putative, expressed	3.101763021
LOC_Os01g50570	N		cytochrome P450 monooxygenase CYP711A12, putative, expressed	2.603583291
LOC_Os07g23570	N		cytochrome P450 72A1, putative, expressed	2.794491382
LOC_Os04g38360	N		wound/stress protein, putative, expressed	6.223558757
LOC_Os01g43890	N		OsSCP4 - Putative Serine Carboxypeptidase homologue, expressed	2.104385548
LOC_Os05g27580	N		wound-induced protein WI12, putative, expressed	2.297068722
LOC_Os02g36300	N		zinc finger, C3HC4 type domain containing protein, expressed	2.815405937
LOC_Os04g46820	N		LTPL121 - Protease inhibitor/seed storage/LTP family protein precursor, putative, expressed	2.583613475
LOC_Os01g70540	N		retrotransposon protein, putative, unclassified, expressed	2.395987989
LOC_Os02g25780	N		expressed protein	2.107541107
LOC_Os01g70970	N		protein kinase, putative, expressed	3.791315945
LOC_Os01g64540	N		anthocyanidin 3-O-glucosyltransferase, putative, expressed	3.901681652
LOC_Os03g62330	N		expressed protein	2.637671537

LOC_Os10g21670	N		dehydration stress-induced protein, putative, expressed	3.622301067
LOC_Os01g16310	N		glycosyl hydrolase, family 31, putative, expressed	16.34506158
LOC_Os06g04850	N		homeobox associated leucine zipper, putative, expressed	2.008641669
LOC_Os03g43970	N		amino acid permease family protein, putative, expressed	2.810957121
LOC_Os10g19200	N		vegetative cell wall protein gp1 precursor, putative, expressed	2.285761681
LOC_Os07g44860	N		gibberellin receptor GID1L2, putative, expressed	2.467614394
LOC_Os01g61080	N	OsWRKY24	WRKY24, expressed	2.019287674
LOC_Os05g12580	N		expressed protein	2.235256925
LOC_Os11g24374	N		OsSCP55 - Putative Serine Carboxypeptidase homologue, expressed	2.208265249
LOC_Os01g50030	N		CPuORF25 - conserved peptide uORF-containing transcript, expressed	2.372254085
LOC_Os06g11290	N	OsOPR1	12-oxophytodienoate reductase, putative, expressed	3.124698233

Supplemental Table 5. Cytokinin-inducible genes regulated by *WOX11*

Down-regulated gene locus	With motif or not	Annotation	Fold Change
LOC_Os01g03330	Y	BBTI3 - Bowman-Birk type bran trypsin inhibitor precursor, expressed	0.451
LOC_Os01g04330	N	OsCML16 - Calmodulin-related calcium sensor protein, expressed	0.492
LOC_Os01g09700	N	aminotransferase, classes I and II, domain containing protein, expressed	0.409
LOC_Os01g24600	Y	fruit bromelain precursor, putative, expressed	0.359
LOC_Os01g38229	Y	peptidyl-prolyl isomerase, putative, expressed	0.386
LOC_Os01g47070	Y	glycosyl hydrolase, putative, expressed	0.301
LOC_Os01g51570	N	glycosyl hydrolases family 17, putative, expressed	0.472
LOC_Os01g71710	N	amino acid permease family protein, putative, expressed	0.307
LOC_Os02g12080	Y	expressed protein	0.344
LOC_Os02g13510	N	receptor-like protein kinase 5 precursor, putative, expressed	0.499
LOC_Os02g43170	N	B-box zinc finger family protein, putative, expressed	0.159
LOC_Os02g52180	N	plastocyanin-like domain containing protein, putative, expressed	0.479
LOC_Os03g03034	Y	flavonol synthase/flavanone 3-hydroxylase, putative, expressed	0.330
LOC_Os03g08320	N	ZIM domain containing protein, putative, expressed	0.498
LOC_Os03g09980	N	sulfate transporter, putative, expressed	0.408
LOC_Os03g10210	Y	homeobox domain containing protein, expressed	0.353
LOC_Os03g13520	N	endonuclease/exonuclease/phosphatase family domain containing protein, expressed	0.367
LOC_Os03g19670	N	GDSL-like lipase/acylhydrolase, putative, expressed	0.449
LOC_Os03g20120	Y	glycosyl transferase 8 domain containing protein, putative, expressed	0.309
LOC_Os03g22200	N	nodulin MtN3 family protein, putative, expressed	0.318
LOC_Os03g46860	Y	helix-loop-helix DNA-binding protein, putative, expressed	0.276

LOC_Os03g49350	N	lipozygenase protein, putative, expressed	0.470
LOC_Os04g01690	Y	pyridoxal-dependent decarboxylase protein, putative, expressed	0.194
LOC_Os04g10750	N	inorganic phosphate transporter, putative, expressed	0.453
LOC_Os04g34600	N	abscisic stress-ripening, putative, expressed	0.321
LOC_Os04g45270	N	aspartyl protease family protein, putative, expressed	0.439
LOC_Os04g56950	N	jasmonate O-methyltransferase, putative, expressed	0.385
LOC_Os05g05060	N	expressed protein	0.372
LOC_Os05g10370	Y	acid phosphatase, putative, expressed	0.429
LOC_Os05g28520	N	MRH1, putative, expressed	0.484
LOC_Os06g03810	N	expressed protein	0.224
LOC_Os06g11650	N	phosphate-induced protein 1 conserved region domain containing protein, expressed	0.464
LOC_Os06g32160	N	THION7 - Plant thionin family protein precursor, expressed	0.063
LOC_Os06g32990	N	peroxidase precursor, putative, expressed	0.489
LOC_Os06g41740	N	transposon protein, putative, CACTA, En/Spm sub-class, expressed	0.418
LOC_Os06g44930	Y	expressed protein	0.466
LOC_Os06g49660	N	transferase family protein, putative, expressed	0.418
LOC_Os07g23430	Y	fatty acid desaturase, putative, expressed	0.385
LOC_Os07g38170	Y	remorin, putative, expressed	0.458
LOC_Os07g39880	N	retrotransposon, putative, centromere-specific, expressed	0.490
LOC_Os08g07180	Y	cytokinin-O-glucosyltransferase 2, putative, expressed	0.454
LOC_Os08g34280	Y	cinnamoyl-CoA reductase, putative, expressed	0.466
LOC_Os08g34984	N	DUF581 domain containing protein, expressed	0.353
LOC_Os08g35210	Y	ferric reductase, putative, expressed	0.435
LOC_Os08g41880	Y	nucleotide pyrophosphatase/phosphodiesterase, putative, expressed	0.318
LOC_Os09g23140	Y	endonuclease/exonuclease/phosphatase family domain containing protein, expressed	0.444

LOC_Os09g24370	N	expressed protein	0.343
LOC_Os09g28210	N	bHelix-loop-helix transcription factor, putative, expressed	0.255
LOC_Os09g31430	N	Os9bglu30 - beta-glucosidase, similar to Os4bglu12 exoglucanase, expressed	0.417
LOC_Os10g39680	Y	CHIT14 - Chitinase family protein precursor, expressed	0.358
LOC_Os11g02130	Y	peroxidase precursor, putative, expressed	0.463
LOC_Os11g04409	Y	expressed protein	0.434
LOC_Os11g05740	N	B3 DNA binding domain containing protein, expressed	0.314
LOC_Os11g12530	N	receptor-like protein kinase 5 precursor, putative, expressed	0.472
LOC_Os11g20160	N	O-methyltransferase, putative, expressed	0.499
LOC_Os12g08090	Y	amino acid transporter, putative, expressed	0.329
LOC_Os12g08130	Y	amino acid transporter, putative, expressed	0.248
LOC_Os12g36240	Y	inhibitor I family protein, putative, expressed	0.395

Up-regulated gene locus	With motif or not	Annotation	Fold Change
LOC_Os01g50030	Y	CPuORF25 - conserved peptide uORF-containing transcript, expressed	2.372
LOC_Os01g74020	N	MYB family transcription factor, putative, expressed	2.650
LOC_Os06g10100	N	expressed protein	2.122
LOC_Os10g03390	N	transferase family protein, putative, expressed	4.711
LOC_Os10g37340	Y	cystathionine gamma-synthase, putative, expressed	6.008
LOC_Os10g40480	N	LTPL143 - Protease inhibitor/seed storage/LTP family protein precursor, expressed	4.803
LOC_Os11g31770	Y	expressed protein	2.734

Supplemental Table 6. Stress related differentially expressed genes

Down-regulated gene locus	With motif or not	Annotation	Fold change
LOC_Os06g32160	N	THION7 - Plant thionin family protein precursor, expressed	0.063333
LOC_Os05g04490	Y	peroxidase precursor, putative, expressed	0.105476
LOC_Os03g22590	Y	nodulin MtN3 family protein, putative, expressed	0.111777
LOC_Os12g12600	Y	dirigent, putative, expressed	0.125658
LOC_Os10g18820	N	dirigent, putative, expressed	0.154301
LOC_Os09g27820	N	1-aminocyclopropane-1-carboxylate oxidase protein, putative, expressed	0.156338
LOC_Os07g01410	N	peroxidase precursor, putative, expressed	0.185569
LOC_Os04g01690	Y	pyridoxal-dependent decarboxylase protein, putative, expressed	0.193768
LOC_Os06g14240	N	hsp20/alpha crystallin family protein, putative, expressed	0.212333
LOC_Os08g16910	N	dehydrogenase, putative, expressed	0.212657
LOC_Os10g34040	N	nodulin, putative, expressed	0.224737
LOC_Os01g71380	N	glycosyl hydrolases family 17, putative, expressed	0.257565
LOC_Os10g02300	N	uncharacterized Cys-rich domain containing protein, putative, expressed	0.260474
LOC_Os04g57130	N	dirigent-like protein pDIR17, putative, expressed	0.261672
LOC_Os07g48030	Y	peroxidase precursor, putative, expressed	0.262527
LOC_Os01g72910	N	abscisic stress-ripening, putative, expressed	0.263295
LOC_Os11g28470	N	expressed protein	0.265527
LOC_Os04g37490	N	oxidoreductase, aldo/keto reductase family protein, putative, expressed	0.269338
LOC_Os07g44370	N	dirigent, putative, expressed	0.269497
LOC_Os10g18760	N	dirigent, putative, expressed	0.270146
LOC_Os09g01290	N	hhH-GPD superfamily base excision DNA repair protein, putative, expressed	0.270716

LOC_Os03g15960	N	hsp20/alpha crystallin family protein, putative, expressed	0.275765
LOC_Os05g06970	Y	peroxidase precursor, putative, expressed	0.294982
LOC_Os01g47070	Y	glycosyl hydrolase, putative, expressed	0.301166
LOC_Os03g13210	N	peroxidase precursor, putative, expressed	0.305256
LOC_Os05g07710	N	WD40-like domain containing protein, putative, expressed	0.305635
LOC_Os06g13210	N	peptide transporter PTR2, putative, expressed	0.308205
LOC_Os03g20120	Y	glycosyl transferase 8 domain containing protein, putative, expressed	0.30899
LOC_Os03g03910	N	catalase domain containing protein, expressed	0.312743
LOC_Os04g01740	N	heat shock protein, putative, expressed	0.313166
LOC_Os09g31482	Y	splicing factor U2AF, putative, expressed	0.314875
LOC_Os02g33820	N	abscisic stress-ripening, putative, expressed	0.31819
LOC_Os07g05720	Y	TCP family transcription factor, putative, expressed	0.214914
LOC_Os03g10210	Y	homeobox domain containing protein, expressed	0.353078
LOC_Os04g34600	N	abscisic stress-ripening, putative, expressed	0.321128
LOC_Os03g47810	N	nodulin family protein, putative, expressed	0.325775
LOC_Os03g03034	N	flavonol synthase/flavanone 3-hydroxylase, putative, expressed	0.329728
LOC_Os06g33100	N	peroxidase precursor, putative, expressed	0.334134
LOC_Os12g04340	Y	sulfotransferase domain containing protein, expressed	0.335141
LOC_Os01g64470	N	harpin-induced protein 1 domain containing protein, expressed	0.340824
LOC_Os10g39680	Y	CHIT14 - Chitinase family protein precursor, expressed	0.357747
LOC_Os01g24600	N	fruit bromelain precursor, putative, expressed	0.359215
LOC_Os09g26960	N	cytochrome P450, putative, expressed	0.361679
LOC_Os09g35800	N	UDP-glucose 4-epimerase, putative, expressed	0.373528
LOC_Os01g46760	Y	protein phosphatase 2C, putative, expressed	0.376552
LOC_Os01g43530	N	late embryogenesis abundant protein, putative, expressed	0.380158

LOC_Os10g33170	N	POT domain containing peptide transporter, putative, expressed	0.384869
LOC_Os01g24550	N	fruit bromelain precursor, putative, expressed	0.385997
LOC_Os03g13160	N	non-symbiotic hemoglobin 2, putative, expressed	0.390756
LOC_Os12g36240	N	inhibitor I family protein, putative, expressed	0.395448
LOC_Os07g48020	N	peroxidase precursor, putative, expressed	0.401572
LOC_Os03g09980	N	sulfate transporter, putative, expressed	0.407745
LOC_Os02g53750	N	tyrosine protein kinase domain containing protein, putative, expressed	0.411225
LOC_Os05g33644	Y	inosine-uridine preferring nucleoside hydrolase family protein, putative, expressed	0.414887
LOC_Os03g14180	N	hsp20/alpha crystallin family protein, putative, expressed	0.42011
LOC_Os07g34670	Y	peroxidase precursor, putative, expressed	0.427214
LOC_Os08g40680	Y	glycosyl hydrolase, putative, expressed	0.427258
LOC_Os02g55890	N	inorganic H+ pyrophosphatase, putative, expressed	0.430111
LOC_Os08g35210	N	ferric reductase, putative, expressed	0.435499
LOC_Os12g28100	N	NBS-LRR disease resistance protein, putative, expressed	0.436127
LOC_Os02g08490	N	chaperone protein clpB 1, putative, expressed	0.436529
LOC_Os06g16300	N	BRASSINOSTEROID INSENSITIVE 1-associated receptor kinase 1 precursor, putative, expressed	0.437705
LOC_Os02g02400	Y	catalase isozyme A, putative, expressed	0.439712
LOC_Os04g41980	N	ATOZI1, putative, expressed	0.440355
LOC_Os01g71820	N	glycosyl hydrolases family 17, putative, expressed	0.441079
LOC_Os11g02080	N	expressed protein	0.44148
LOC_Os03g25330	N	peroxidase precursor, putative, expressed	0.446236
LOC_Os12g02040	N	hypoxia-responsive family protein, putative, expressed	0.458794
LOC_Os02g54140	N	hsp20/alpha crystallin family protein, putative, expressed	0.462171
LOC_Os11g02130	Y	peroxidase precursor, putative, expressed	0.463159
LOC_Os08g34280	Y	cinnamoyl-CoA reductase, putative, expressed	0.465596

LOC_Os09g07450	N	flavonol synthase, putative, expressed	0.468448
LOC_Os12g42810	N	mov34/MPN/PAD-1 family protein, expressed	0.469713
LOC_Os01g51570	N	glycosyl hydrolases family 17, putative, expressed	0.472272
LOC_Os11g07690	N	dirigent, putative, expressed	0.472773
LOC_Os02g38392	N	NBS-LRR disease resistance protein, putative, expressed	0.472865
LOC_Os03g55530	N	HLS, putative, expressed	0.478536
LOC_Os11g02440	N	chalcone--flavonone isomerase, putative, expressed	0.480264
LOC_Os03g52860	N	lipoxygenase, putative, expressed	0.481978
LOC_Os04g41410	N	expressed protein	0.48684
LOC_Os06g32990	N	peroxidase precursor, putative, expressed	0.488876
LOC_Os01g45140	N	anthocyanin 3-O-beta-glucosyltransferase, putative, expressed	0.488971
LOC_Os01g04330	N	OsCML16 - Calmodulin-related calcium sensor protein, expressed	0.492362
LOC_Os08g37250	N	patatin, putative, expressed	0.492819
LOC_Os04g28210	N	verticillium wilt disease resistance protein, putative, expressed	0.493072
LOC_Os12g41680	Y	No apical meristem protein, putative, expressed	0.402189
LOC_Os03g29410	Y	tyrosine protein kinase domain containing protein, putative, expressed	0.495353
LOC_Os04g45480	N	heat shock protein STI, putative, expressed	0.495649
LOC_Os03g44380	N	9-cis-epoxycarotenoid dioxygenase 1, chloroplast precursor, putative, expressed	0.497156
LOC_Os03g08320	N	ZIM domain containing protein, putative, expressed	0.498108

Up-regulated gene locus	With motifor not	Annotation	Fold change
LOC_Os12g10340	Y	NBS-LRR type resistance protein, putative, expressed	12.37035
LOC_Os07g48060	N	peroxidase precursor, putative, expressed	9.229174
LOC_Os10g40470	N	LTPL142 - Protease inhibitor/seed storage/LTP family protein precursor, putative, expressed	7.866752
LOC_Os11g26750	N	dehydrin, putative, expressed	7.837323

LOC_Os06g16790	Y	go35 NBS-LRR, putative, expressed	6.819676
LOC_Os10g40480	N	LTPL143 - Protease inhibitor/seed storage/LTP family protein precursor, expressed	4.802609
LOC_Os03g22010	Y	peroxidase precursor, putative, expressed	3.792859
LOC_Os10g38720	Y	glutathione S-transferase, putative, expressed	3.137664
LOC_Os06g11290	Y	12-oxophytodienoate reductase, putative, expressed	3.124698
LOC_Os03g44740	Y	cytochrome P450, putative, expressed	3.075155
LOC_Os03g05910	Y	expressed protein	2.938844
LOC_Os06g29470	Y	peroxidase precursor, putative, expressed	2.836298
LOC_Os02g42640	N	CBS domain-containing protein, putative, expressed	2.813498
LOC_Os01g49240	N	limonoid UDP-glucosyltransferase, putative, expressed	2.762529
LOC_Os07g12670	N	expressed protein	2.683725
LOC_Os07g12680	N	LZ-NBS-LRR class RGA, putative, expressed	2.683725
LOC_Os01g41430	Y	UDP-glucoronosyl and UDP-glucosyl transferase, putative, expressed	2.605497
LOC_Os03g07510	N	expressed protein	2.523218
LOC_Os05g42350	Y	ferredoxin--nitrite reductase, putative, expressed	2.450637
LOC_Os11g43700	Y	RGH1A, putative, expressed	2.429681
LOC_Os10g34020	N	glutathione S-transferase, putative, expressed	2.422432
LOC_Os12g17480	Y	MLA12, putative, expressed	2.342214
LOC_Os03g19720	N	EF hand family protein, putative, expressed	1.976056
LOC_Os04g46970	Y	glucosyltransferase, putative, expressed	2.240803
LOC_Os02g39330	N	CHIT1 - Chitinase family protein precursor, expressed	2.194433
LOC_Os04g44470	N	KUN1 - Kunitz-type trypsin inhibitor precursor, expressed	2.160361
LOC_Os10g26460	N	bHLH family protein, putative, expressed	2.125051
LOC_Os03g09970	Y	sulfate transporter, putative, expressed	2.116969
LOC_Os10g38150	N	glutathione S-transferase, N-terminal domain containing protein, expressed	2.086533

LOC_Os10g20450	N	MATE efflux family protein, putative, expressed	2.060882
LOC_Os03g02939	N	peroxidase precursor, putative, expressed	2.040954
LOC_Os01g45110	N	anthocyanin 3-O-beta-glucosyltransferase, putative, expressed	2.029371
LOC_Os01g54890	Y	ethylene-responsive transcription factor 2, putative, expressed	3.101763
LOC_Os02g41904	Y	DEF7 - Defensin and Defensin-like DEFL family, expressed	2.008733

Supplemental Table 7. Metabolism enrichment GO

Regulated gene locus	With motif or not	GO name	Reported name	Fold change
LOC_Os01g72910	N	response to stress	*Asr2; ASR4; OsASR6*	0.263295
LOC_Os02g33820	Y	response to stress	*Asr3; OsASR3; ASR1*	0.318189
LOC_Os02g54140	Y	response to stress		0.462172
LOC_Os03g14180	N	response to stress	*Oshsp26*	0.420111
LOC_Os03g15960	N	response to stress		0.275764
LOC_Os03g16920	Y	response to stress		0.439126
LOC_Os03g53340	N	response to stress	*OsHsfA2a*	0.287179
LOC_Os04g01740	N	response to stress		0.313167
LOC_Os04g34600	N	response to stress	*Asr4; ASR6; OsASR2*	0.321127
LOC_Os06g11290	N	response to stress	*OsOPR1*	3.124698
LOC_Os06g14240	N	response to stress		0.212333
LOC_Os09g35790	N	response to stress	*OsHsfB2c*	0.338428
LOC_Os11g26750	N	response to stress		7.837323
LOC_Os01g43774	Y	oxidation reduction		0.469288
LOC_Os01g50530	N	oxidation reduction		2.067259
LOC_Os01g50580	N	oxidation reduction	*SLB2*	2.603583
LOC_Os01g51860	Y	oxidation reduction		0.314395
LOC_Os01g70540	N	oxidation reduction		2.395988
LOC_Os02g01890	Y	oxidation reduction		0.413001
LOC_Os02g02400	Y	oxidation reduction	*OsCATA*	0.439709
LOC_Os02g21810	N	oxidation reduction		3.926823
LOC_Os03g02939	N	oxidation reduction		2.040954

LOC_Os03g03910	N	oxidation reduction	*OsCATC;noe1*	0.312744
LOC_Os03g04680	N	oxidation reduction	*OsCYP96B4; OsDSS1; sd37; bsh1*	0.345122
LOC_Os03g13210	N	oxidation reduction		0.305255
LOC_Os03g22010	N	oxidation reduction		3.792859
LOC_Os03g25330	N	oxidation reduction		0.446238
LOC_Os03g44740	N	oxidation reduction		3.075155
LOC_Os03g49350	N	oxidation reduction	*OsLOX4*	0.470037
LOC_Os03g52860	N	oxidation reduction	*L-2*	0.481977
LOC_Os03g59100	N	oxidation reduction		2.103895
LOC_Os04g04770	N	oxidation reduction		3.228668
LOC_Os04g15920	N	oxidation reduction		0.439673
LOC_Os04g37490	N	oxidation reduction		0.26934
LOC_Os05g04490	Y	oxidation reduction		0.105475
LOC_Os05g06970	Y	oxidation reduction		0.294983
LOC_Os06g29470	N	oxidation reduction		2.836298
LOC_Os06g32990	N	oxidation reduction		0.488878
LOC_Os06g33100	Y	oxidation reduction		0.334132
LOC_Os07g01410	Y	oxidation reduction		0.185569
LOC_Os07g07440	N	oxidation reduction		2.157196
LOC_Os07g23570	N	oxidation reduction		2.794491
LOC_Os07g32570	N	oxidation reduction		4.675741
LOC_Os07g34670	Y	oxidation reduction		0.427213
LOC_Os07g37420	N	oxidation reduction		0.280513
LOC_Os07g48020	Y	oxidation reduction		0.401572
LOC_Os07g48030	Y	oxidation reduction		0.262527

LOC_Os07g48060	N	oxidation reduction		9.229174
LOC_Os08g01510	Y	oxidation reduction		0.377787
LOC_Os08g16910	N	oxidation reduction		0.212657
LOC_Os08g35210	Y	oxidation reduction	*OsrbohE; Osrboh6*	0.435499
LOC_Os08g36860	Y	oxidation reduction	*OsABA8ox2*	0.489379
LOC_Os09g23820	N	oxidation reduction		2.553159
LOC_Os09g26960	N	oxidation reduction		0.361678
LOC_Os09g27820	N	oxidation reduction	*OsACO1; OsACO2*	0.156338
LOC_Os10g26340	N	oxidation reduction	*PLA1*	0.288169
LOC_Os10g31780	N	oxidation reduction		4.147934
LOC_Os10g36980	N	oxidation reduction		2.0643
LOC_Os11g02130	Y	oxidation reduction		0.46316
LOC_Os11g14910	N	oxidation reduction		0.248551
LOC_Os12g12590	Y	oxidation reduction		0.277789
LOC_Os11g02440	N	flavonoid biosynthetic process		0.480264
LOC_Os12g02370	N	flavonoid biosynthetic process		0.497093
LOC_Os01g47070	Y	carbohydrate metabolic process		0.301164
LOC_Os02g39330	N	carbohydrate metabolic process		2.194433
LOC_Os04g09390	N	chitin catabolic process		0.397567
LOC_Os10g39680	Y	carbohydrate metabolic process		0.357747
LOC_Os04g46810	N	lipid transport		2.091987
LOC_Os04g46820	N	lipid transport		2.583613
LOC_Os10g20840	N	lipid transport		0.313038
LOC_Os10g40460	N	lipid transport		3.809346
LOC_Os10g40470	N	lipid transport		7.866752
LOC_Os10g40480	N	lipid transport		4.802609

LOC_Os01g16310	N	carbohydrate metabolic process		16.34506
LOC_Os01g51570	N	carbohydrate metabolic process		0.472272
LOC_Os01g71380	N	carbohydrate metabolic process		0.257565
LOC_Os01g71820	Y	carbohydrate metabolic process		0.441081
LOC_Os02g10300	N	carbohydrate metabolic process		0.41468
LOC_Os02g17900	N	carbohydrate metabolic process		0.475252
LOC_Os05g09500	N	carbohydrate metabolic process	*OsHXK7*	2.008142
LOC_Os05g20020	Y	carbohydrate metabolic process		0.430197
LOC_Os06g46340	Y	carbohydrate metabolic process		0.340786
LOC_Os07g35560	N	carbohydrate metabolic process		0.205874
LOC_Os08g13980	N	carbohydrate metabolic process		2.79328
LOC_Os08g40680	Y	carbohydrate metabolic process		0.427259
LOC_Os09g31410	N	carbohydrate metabolic process		0.228296
LOC_Os09g31430	N	carbohydrate metabolic process		0.417194
LOC_Os09g35800	N	carbohydrate metabolic process		0.373529
